# Drivers of biomass stocks and productivity of tropical secondary forests

**DOI:** 10.1002/ecy.4488

**Published:** 2024-12-04

**Authors:** Tomonari Matsuo, Lourens Poorter, Masha T. van der Sande, Salim Mohammed Abdul, Dieudonne Wedaga Koyiba, Justice Opoku, Bas de Wit, Tijs Kuzee, Lucy Amissah

**Affiliations:** ^1^ Forest Ecology and Forest Management Group Wageningen University Wageningen the Netherlands; ^2^ CSIR‐Forestry Research Institute of Ghana Kumasi Ghana

**Keywords:** biomass stocks and productivity, fine roots, forest structure, functional trait composition, macroclimate, soil carbon and nutrients, species diversity, tropical secondary forest

## Abstract

Young tropical secondary forests play an important role in the local and global carbon cycles because of their large area and rapid biomass accumulation rates. This study examines how environmental conditions and forest attributes shape biomass compartments and the productivity of young tropical secondary forests. We compared 36 young secondary forest stands that differed in the time since agricultural land abandonment (2.3–3.6 years) from dry and wet regions in Ghana. We quantified biomass stocks in living and dead stems, roots, and soil, and aboveground biomass and litter productivity. We used structural equation models to evaluate how macroclimate, soil nutrients (N, P), and forest attributes (structure, diversity, and functional composition) affect ecosystem functioning. After three years of succession, tropical wet forests stored on average 115 t biomass ha^−1^ (the sum of aboveground living and dead biomass, belowground fine root biomass, and soil organic matter), and dry forests stored 99 t ha^−1^. These values represent 31% (in the wet forest) and 39% (in the dry forest) of the biomass compared with neighboring old‐growth forests. The majority of forest ecosystem biomass was stored in the soil (70%) and aboveground living vegetation (25%). Macroclimate strongly shaped forest attributes, which in turn determined biomass stocks and productivity. Soil phosphorus strongly increased litter production and soil organic matter, confirming that it is a limiting element in tropical ecosystems. Tree density and species diversity increased forest biomass stocks, suggesting crown packing and complementary resource use enhance forest functioning. A more conservative trait composition (high wood density) increased biomass stocks but reduced productivity, indicating that quantity, identity, and quality of species affect ecosystem functioning.

## INTRODUCTION

Tropical forests store around 25% of the global terrestrial carbon (Bonan, 2008) and account for 34% of terrestrial gross primary productivity (Beer et al., 2010), which makes them important for climate change mitigation. Despite their importance, over half of the world's tropical old‐growth forests have been deforested for human activities such as crop cultivation or cattle ranching (IPBES, 2019; Keenan et al., 2015). In these previously deforested areas, secondary succession leads to a rapid accumulation of biomass in the vegetation and soil (Jones et al., 2019; Martin et al., 2013). These biomass accumulation rates during succession vary strongly with macroclimate and soil conditions (Poorter et al., 2016) because they reflect initial environmental conditions (e.g., resource availability) as they grow, and initial forest attributes (e.g., forest structure and species composition). These environmental conditions and forest attributes in early succession, therefore, determine the large variations in the speed and direction of successional pathways (Meiners et al., 2015; van Breugel et al., 2019). Here, we examine how environmental conditions and forest attributes determine different compartments of biomass pools and productivity in young tropical forests.

In old‐growth tropical forests, approximately half of the carbon is stored in the aboveground living compartments, around 10% in the belowground root biomass, less than 10% in the dead organic matter, and 30%–40% in the soil (Malhi et al., 2009). In contrast, in young tropical secondary forests, less carbon is stored in the aboveground vegetation and relatively more in the belowground roots and soil because slash‐and‐burn agriculture has led to aboveground biomass (AGB) removal, and the vegetation is still developing (Jones et al., 2019). This highlights the importance of studying not only AGB but also other biomass pools to get a comprehensive picture of forest carbon recovery during succession.

Structural, taxonomic, and functional attributes of the forest determine forest biomass pools and productivity through various mechanisms (Finegan et al., 2015; Poorter et al., 2017). Structural attributes, for example, high stand basal area or tree density, are associated with a large photosynthetically active leaf area and thus high carbon sequestration rates (Lehnebach et al., 2018; Pan et al., 2021). Therefore, they store more carbon in aboveground leaf, branch, and stem biomass, as well as in the belowground root biomass (Kenzo et al., 2009; Poorter, van der Sande, et al., 2015). The regular turnover of these plant organs also leads to a high litter production and build up of soil organic matter (SOM) (Feng et al., 2019).

Taxonomic attributes, for example, species richness and evenness, may increase carbon accumulation and stocks through a high resource capture and use (the “niche complementarity effect,” Tilman, 1999), and through an increasing chance to include a highly productive species (“the sampling effect,” Loreau, 1998). Similarly, increased species richness and evenness can increase belowground biomass through more efficient root‐filling of the soil (Brassard et al., 2013; Lei et al., 2012). However, little is known whether these positive biodiversity–ecosystem functioning relationships can also be observed in young tropical secondary forests that are dominated by few pioneer species that contribute most to ecosystem functioning (Lohbeck et al., 2016).

Functional traits determine species' performance in terms of recruitment, growth, and survival (Matsuo, Martínez‐Ramos, et al., 2024; Violle et al., 2007). Therefore, they also determine ecosystem functioning (Lohbeck et al., 2015; Yuan et al., 2018). Because the dominant species in the community often drive ecosystem functioning (mass‐ratio hypothesis, Grime, 1998), dominance‐weighted functional attributes (e.g., community‐weighted mean [CWM] traits) frequently influence carbon accumulation and stocks (Teixeira et al., 2020; Yuan et al., 2019). Hence, functional attributes, such as high CWM leaf nitrogen concentration (LNC) or low CWM wood density (WD), increase carbon accumulation and stocks because of faster biomass growth rates (e.g., Finegan et al., 2015). These species with high LNC or low WD often have short leaf and plant lifespans (Chazdon et al., 2007; Wright et al., 2004); thus, increasing their dominance enhances the production of deadwood and litterfall which, in turn, increases carbon accumulation in the soil (Morriën et al., 2017; Odum, 1969).

Carbon accumulation rates and stocks also vary with environmental conditions (van der Sande, Arets, et al., 2017; van der Sande, Peña‐Claros, et al., 2017). For instance, in wetter, warmer, and more fertile soil conditions, soil microbes are more abundant and active, resulting in faster litter decomposition rates and thus higher soil organic carbon concentrations (Camenzind et al., 2018). In drier and more infertile soil conditions, species allocate more biomass to their (fine) roots to increase water and nutrient uptake (Freschet et al., 2021; van der Sande, Arets, et al., 2017). Besides these direct effects, macroclimate and soil fertility also indirectly affect forest biomass by shaping forest attributes, such as stand basal area, tree density, and species richness, through different species pools, and length and conditions of the growing season (Poorter, van der Sande, et al., 2015; Rozendaal et al., 2019).

**FIGURE 1:**
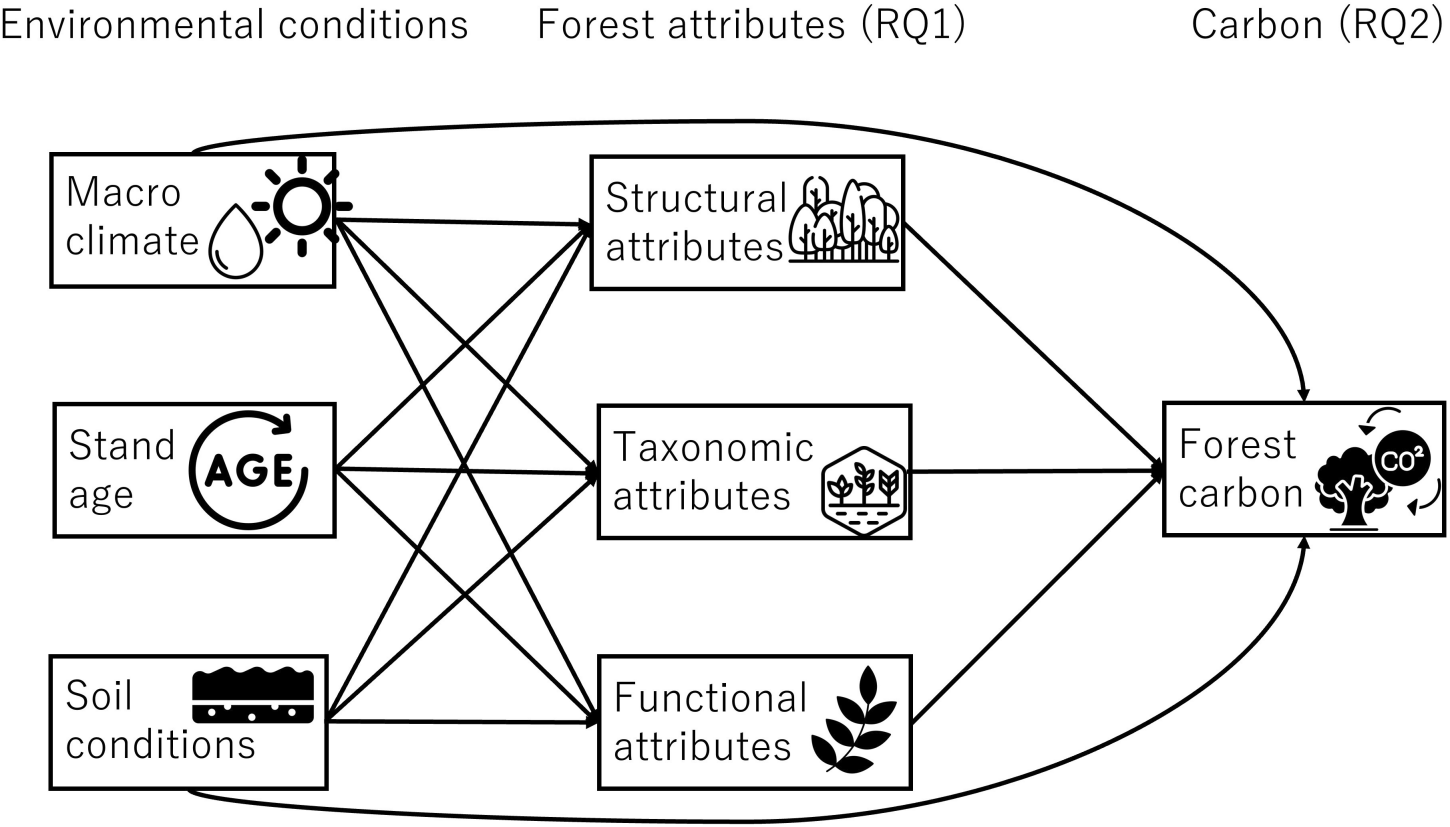
Conceptual model showing how environmental conditions affect forest attributes (Research Question 1), and how environmental conditions and forest attributes together affect forest biomass stocks and productivity (aboveground living biomass, aboveground dead biomass, fine root biomass, soil organic matter, aboveground biomass productivity, and litter production, Research Question 2). Clipart images from Flaticon.com.

Here, we examine how environmental conditions and forest attributes determine different compartments of forest biomass pools and productivity in young tropical forests on abandoned agricultural fields in Ghana (Figure 1). We address two research questions and their corresponding hypotheses:

1. How are forest (1) structural, (2) taxonomic, and (3) functional attributes driven by macroclimate and soil nutrients during early succession? We hypothesize that: (1) stand basal area, maximum stem diameter, and tree density are higher in wet forests and on fertile soils because of more productive environmental conditions, as well as in older forests due to continuous tree recruitment and growth over time; (2) species richness is higher in wet forests and on fertile soils because of more suitable conditions for plants, as well as in older forests because of continuous arrival and recruitment of new species over time; (3) species with acquisitive trait values are more dominant in wet and younger forests and on fertile soils because of more productive environmental conditions.
2. How do environmental conditions and forest attributes determine forest biomass pools and productivity? We hypothesize that forest biomass pools and productivity increase with: (1) climatic wetness and soil fertility because of increased recruitment and growth rates; (2) structural attributes, such as stand basal area, because of a larger photosynthetically active leaf area; (3) species richness because of more efficient resource use and sampling effects; and (4) dominance of more acquisitive species as they grow faster.

## MATERIALS AND METHODS

### Study site

#### Tropical dry region

Research was carried out close to the town of Abofour in the Ashanti region of Ghana (7°11′ N, 1°73′ W). Mean annual precipitation is 1290 mm/year and dry season precipitation (November/December–February) is 28 mm/month (Amissah et al., 2018). Mean monthly maximum temperature is 30.6°C, and mean monthly minimum temperature is 21.2°C (Amissah et al., 2018). The soil is sandy loam with patches of clay (Forestry Division, 1963) and a pH of 5.6–7.8. The forest is classified as a tropical dry semideciduous forest (Hall & Swaine, 1981).

#### Tropical wet region

Research was carried out close to the town of Pataho in the Western region of Ghana (5°10′ N, 2°02′ W). Mean annual precipitation is 1808 mm/year and dry season precipitation (November/December–February) is 82.6 mm/month (Amissah et al., 2018). Mean monthly maximum temperature is 32.0°C and mean monthly minimum temperature is 22.8°C (Amissah et al., 2018). The area is characterized by undulating hills, ranging between 71 and 148 m above sea level. The soil consists of sandy loam with patches of clay (Forestry Division, 1963) and is acidic (pH 4.1–5.6). The forest is classified as a tropical wet/moist evergreen forest (Hall & Swaine, 1981).

In 2021, we established 20 secondary forest plots (25 × 25 m) on recently abandoned maizefields (0 or 1 year since agricultural abandonment) in dry forests and 19 secondary forest plots on abandoned cassava fields in wet forests (Matsuo et al., 2023). During the monitoring, three plots in dry sites were burned and cleared, and thus we excluded them from the analysis (i.e., *N* = 17 in dry forests). Fallow age was determined based on interviews with the landowners and field observations. In 2021, 2022, and 2023, all woody individuals thicker than 1 cm stem dbh were identified to species level, and their dbh and height were measured. Height was measured with a telescoping rod. However, the height data were not used in this study. For multistemmed individuals, we counted the number of stems and measured the dbh of the largest and average stems. We calculated the tree's basal area as π × (dbh/2)^2^. For individuals with multiple stems, we multiplied the number of other stems with their average basal area and added the basal area of the largest stem.

### AGB and productivity

AGB of shrub and tree species was estimated with an allometric formula developed for the young secondary forests in Ghana using dbh and WD (Equation 1; Appendix S1). AGB of liana species was estimated with the existing allometric equation for liana species in tropical secondary forests in Ghana (Equation 2; Addo‐Fordjour & Rahmad, 2013). When the WD data for some species were not available, we used local WD data at the highest taxonomic resolution available (genus‐level or family‐level), or the average WD for each site.

(1) $$\mathrm{AGB}\_\text{tree}\_\text{shrub}=\exp.\left[-1.65+2.14\times \ln\left(\mathrm{dbh}\right)+0.45\times \ln\left(\mathrm{WD}\right)\right]\;\left(r^{2}=0.94\right).$$

(2) $$\mathrm{AGB}\_\text{liana}=-0.36+1.9\times \mathrm{dbh}\;\left(r^{2}=0.99\right).$$

Aboveground living biomass per plot (AGB_living_, in tons per hectare) for each year was calculated by summing the biomass of all live trees in 2022 or 2023 and then multiplying by 16 to express it per hectare. AGB productivity (in tons per hectare per year) was calculated as the difference in AGB_living_ over a one‐year interval. For one plot in the wet forests, we could not calculate biomass productivity because the census was not conducted in 2022.

### Aboveground dead biomass

Deadwood lying on the ground (≥5 cm diameter) was inventoried along two parallel 25‐m‐long transects within each plot that were spaced 15 m apart, using the line‐intercept method. The diameter of all deadwoods bisecting a transect was recorded, along with its diameter at midpoint and length. All standing deadwood with dbh > 1 cm was inventoried throughout each plot, with diameter and height recorded. To estimate their biomass, their volume was calculated using an equation for lying deadwood (Equation 3; Aghimien et al., 2020) or an equation for standing deadwood (Equation 4) with the default shape coefficient (*f* = 0.5, Puletti et al., 2019). Then their volume was converted to biomass by multiplying the average WD of each plot and the average decay factor (*F* = 0.8, Hossain et al., 2019) (Equation 5; Neumann et al., 2023).

(3) $$\text{Lying deadwood volume}=\left(\pi {D_{\text{middle}}}^{2}/4\right)\times L,$$

(4) $$\text{Standing deadwood volume}=f\times \left({\text{πdbh}}^{2}/4\right)\times L,$$

(5) $${\mathrm{AGB}}_{\text{dead}}=F\times \text{Deadwood volume}\times \mathrm{WD},$$

where *D*_middle_ is the diameter at the midpoint (in centimeters), *L* is the total length (in meters), and WD is the average WD of each plot (in grams per cubic centimeter) weighted by their basal area. Aboveground lying dead biomass per plot was calculated as the sum of all lying deadwood biomass within the 50 m^2^ (= 25 m^2^ × 2) transect and then multiplied by 200 to express it per hectare. Similarly, aboveground standing dead biomass per plot was calculated as the sum of all standing dead biomass and then multiplied by 16 to express it per hectare. Lastly, total aboveground dead biomass per plot (AGB_dead_, in tons per hectare) was calculated as the sum of aboveground lying and standingdead biomass.

### Litter production rate

The plots were subdivided into four quadrants (12.5 × 12.5 m), and one litter trap (50 × 50 cm, at a height of 1.0 m) was placed at the center of each quadrant (i.e., four traps per plot). To estimate the annual litter production (in tons per hectare per year), litter was collected every month for 7 months (February–August in 2023), which covers two months of a dry season and five months of a wet season, which is considered as a sufficient sampling effort to estimate the annual litter production. Each month, litter samples were collected and separated into leaf materials (leaves and petioles), branches, reproductive parts (flowers, fruits, and seeds), and animal feces (from lizards, bats, and birds that defecated in the traps). Afterward, these components were oven‐dried at 65°C for 48 h and weighed for their dry mass. Because we were only interested in the forest litter production rate, we only used the sum of leaf materials, branches, and reproductive parts for the litter production rate. The weight of litter was summed for each plot and multiplied by 10,000 to express it as tons per hectare.

### Soil nutrients, SOM, and fine root biomass

In 2021, five soil samples (0–15 cm depth) were collected using an auger with a 23 cm diameter and 21 cm length, and they were pooled per plot and analyzed at CSIR‐Soil Research Institute of Ghana for the following: texture (sand, clay, and silt, in percentage); soil pH with a 1:2.5 mixture (soil:water ratio) using a glass electrode (Bante 930) pH meter; total nitrogen including all forms of organic and inorganic nitrogen (N, in milligrams per gram) based on the Kjeldahl digestion and distillation procedure (Bremner & Keeney, 1965); plant‐available phosphorus (P, in micrograms per gram) determined colorimetrically with HCl:NH_4_F mixture (Bray's No. 1 extract) by ascorbic reduction (Bray & Kurtz, 1945; Olsen & Sommers, 1982); and potassium (K, in centimoles per kilogram) determined with 1.0 M ammonium acetate (NH_4_OAc) by flame photometry. To obtain soil bulk density (BD, in grams per cubic centimeter), additional soil samples (0–15 cm depth) were taken at the same locations using a 5‐cm‐diameter soil ring. These samples were oven‐dried at 105°C for up to 120 h and weighed. BD was calculated as the dry soil mass divided by the volume inside the ring (295 cm^3^). The mean BD per plot was then calculated by averaging the value per plot.

In 2023, soil cores were taken with the same soil ring to collect fine roots and estimate the SOM (in percentage) to a depth of 15 cm. Because in many places rocks were present beyond 15 cm soil depth, sampling was limited to this depth. Additionally, this is the depth at which SOM is most strongly affected by litter production (Feng et al., 2019) and thus changes most rapidly during succession (van der Sande et al., 2022). To account for spatial heterogeneity, for each plot, eight samples were taken for fine root biomass and four samples for SOM. SOM was determined by the modified dichromate oxidation method of Walkley–Black (Nelson & Sommers, 1983). Root samples were processed following the standard protocol (Freschet et al., 2021). Root samples were properly washed after soaking them in water for up to 24 h, then sieved with a 0.25‐mm mesh sieve, and oven‐dried at 65°C for 48 h. Samples were then separated into fine (<2 mm diameter) and coarse (>2 mm) roots, which were weighed separately. We only used the data of fine roots for the analysis. Both fine root biomass and SOM were scaled to tons per hectare in the 15 cm topsoil to compare values with AGB_living_ and AGB_dead_ (also in tons per hectare) and to estimate the total biomass stocks. More details about the collection and analysis of SOM and fine root biomass can be found in Appendix S2.

### Leaf and stem traits

To describe dominance‐weighted community functional properties, we followed standardized protocols (Pérez‐Harguindeguy et al., 2013). We measured four leaf traits (LNC [in milligrams per gram], leaf phosphorus concentration [LPC, in milligrams per gram], leaf mass per area [LMA, in grams per square centimeter], and leaf dry matter content [LDMC, in grams per gram]) and one stem trait (WD, in grams per cubic centimeter). LNC and LPC are important for plant metabolism (Ellsworth & Reich, 1996; Evans, 1989). LMA and LDMC are important for leaf defense against biophysical hazards and therefore increase leaf lifespan (Kitajima & Poorter, 2010). WD is important for vertical growth and wood defense (Poorter et al., 2010).

For leaf traits, we measured 65 species in dry forests and 104 species in wet forests that covered on average 95.0% of the basal area in each plot in dry forests (range 81.9%–99.2%) and 98.5% (91.7%–99.9%) in wet forest. For each species, leaf traits were measured for two sunlit leaves of four or five individuals with a height between 1 and 8 m and a diameter at 30 cm height between 1 and 10 cm (Matsuo, van der Sande, et al., 2024), which is a typical size range in early succession. As leaf traits can be highly plastic in response to irradiance (Poorter et al., 2019), we selected all trees under similar “optimal” high‐light growing conditions.

For WD, we collected the data for 77 species in dry forests and 75 species in wet forests, covering on average 97.6% (range 93.7%–99.9%) in dry forests and 97.9% (range 91.2%–99.9%) in wet forests. For each species, WD was measured from three individuals. WD was based on wood cores (4.3 mm diameter), using an increment borer (Haglöf Sweden, Langsele, Sweden), or stem slices for species with small stems. For stem slices, the fresh volume, including the bark, was determined with the water displacement method. WD was calculated as oven‐dried mass (at 80°C for 48 h) divided by the fresh volume. This measurement was taken in the study area for 61 species studied; data on WD for the remaining species were taken from the WD database in Ghana (Djagbletey et al., 2020). More details about trait measurements can be found in Appendix S3.

### Forest attributes

We assessed three types of forest attributes: structural, taxonomic, and functional attributes of the forest. As structural attributes, we calculated tree density (N, in numbers per hectare) indicating how densely packed trees are in a given forest stand; maximum tree size (dbh_max_, in centimeters) expressed as 5th percentile largest dbh of tree individuals since larger trees store and accumulate more above‐ and belowground biomass than smaller individuals (Kenzo et al., 2009; Stephenson et al., 2014); and stand basal area (BA, in square meters per hectare), which is closely related to the total leaf area (Lehnebach et al., 2018; Shinozaki et al., 1964).

We calculated three taxonomic attributes: species richness per plot, rarefied species richness per 150 individuals, and species evenness. Species richness per plot is the absolute number of species and is thus independent of species abundance. Rarefied species richness is the number of species observed when a certain number of trees are randomly drawn from a plot (Chao & Chiu, 2016). Such rarefaction removes the confounding effect of tree density on species richness. Hence, rarefied species richness increases both with absolute species richness and with species evenness per plot (Appendix S4: Table S1), and thus serves as the measure of diversity. For rarefied richness, we used 150 individuals, as this is the minimum number of individuals found in all plots in 2022 and 2023. Species evenness is a measure of how evenly tree species are abundant (Help et al., 1998). We calculated species evenness based on Hill numbers because they have been developed as a mathematically coherent family of indices that only differ by the sensitivity to species' relative abundances (Chao et al., 2014). Hence, species evenness was calculated as the non‐transformed Shannon diversity divided by the absolute species richness per plot.

As functional attributes, we calculated the CWM for each trait (i.e., representing the trait value of an average‐sized tree species in the community), by multiplying each species' trait value by its relative dominance in the plot (in terms of basal area) and then summing all species occurring in the plot (Equation 6; Lohbeck et al., 2015).

(6) $$\mathrm{CWM}=\sum_{i=1}^{s}w_{i}\times x_{i},$$

where *w*_*i*_ is the relative basal area of species *i* based on the total basal area of species with trait data, *x*_*i*_ is the trait value of species *i*, and *S* is the total number of species with trait data.

### Statistical analyses

To understand how environmental conditions and forest attributes determine forest biomass pools and productivity, structural equation modeling (SEM, as implemented in the R package Lavaan, Rosseel, 2012) was used to relate, causally and hierarchically, environmental conditions, forest attributes, and forest biomass pools or productivity. Our a priori conceptual model (see Figure 1) was based on existing knowledge of this study system and previous studies in tropical old‐growth forests (e.g., Finegan et al., 2015; Poorter et al., 2017). Although the variation in stand age is relatively small among plots (less than 1.5 years), we included stand age as an environmental condition because it captures several environmental conditions (e.g., understory irradiance), which might change with vegetation development during succession (Matsuo et al., 2021, 2022). To reduce the number of potential models, we selected two soil variables (soil N and P) based on the standardized effect size and collinearity in a series of linear models. In the models, each forest attribute served as a response variable, with macroclimate, stand age, and six soil variables (sand, pH, soil N, P, K, and BD) as predictor variables. For functional attributes, we used a subset of traits based on their relevance. For AGB_living_, AGB_dead_, and productivity, we used LMA, LNC, LPC, and WD because they determine tree biomass growth rates. Low LMA indicates an efficient biomass investment per unit leaf area to capture light, whereas LNC and LPC increase photosynthetic capacity (Finegan et al., 2015). Low WD increases stem hydraulic conductivity, photosynthetic carbon gain, and volumetric growth capacity, and decreases stem mass (Poorter et al., 2010; Reich, 2014). For litter production, we used LNC, LPC, LMA, and LDMC. LNC and LPC increase leaf turnover rate (Reich, 2014; Wright et al., 2004) and therefore increase litter production rates, while LMA and LDMC increase leaf longevity (Onoda et al., 2017) and therefore reduce leaf abscission and litter production rates. For fine root biomass, we used LNC, LPC, LMA, and LDMC. High LNC and LPC indicate high root nitrogen concentration, which may increase root turnover rate and reduce fine root biomass (Terzaghi et al., 2013; Westoby & Wright, 2006). In contrast, high LMA and LDMC indicate high root tissue density and thus root longevity, which could increase the residual time of fine roots and thus fine root biomass (Kramer‐Walter et al., 2016; Sierra Cornejo et al., 2020). For SOM, we used LNC, LPC, LDMC, and WD. High LNC and LPC can supply nutrients to microbial decomposers and thus facilitate their activities, which could increase decomposition rates and facilitate carbon transfer from living organs and litter to the soil (Enriquez et al., 1993). Meanwhile, high LDMC and WD exhibit strong resistance to decomposer organisms and thus decrease the rates of decomposition and carbon transfer (Freschet et al., 2012).

We made a series of SEMs for each of our six response variables (AGB_living_, AGB_dead_, fine root biomass, SOM, aboveground productivity, and litter production), as they can be driven by different environmental conditions and forest attributes. This produced 72 alternative models for each response variable (2 soil properties × 3 structural attributes × 3 taxonomic attributes × 4 functional attributes). We additionally made a series of SEMs for total biomass stocks (the sum of AGB_living_, AGB_dead_, fine root biomass, and SOM, in tons per hectare), and for total AGB productivity (the sum of AGB productivity and litter production, in tons per hectare per year) to identify the general drivers of biomass stocks and productivity. For model selection, we initially rejected all models with a significantly poor fit (*p* < 0.05 from the chi‐squared test). Subsequently, we selected the best‐fitting models based on the absolute *R* ^2^ of the response because we aimed to understand the drivers of forest biomass pools and productivity. All data analyses were conducted using the statistical package R (version 3.4.0; R Foundation for Statistical Computing, Vienna, Austria).

## RESULTS

After three years of succession, on average, the wet forest exhibited significantly higher values than the dry forest for AGB_living_ (31 vs. 21 t ha^−1^), AGB_dead_ (0.4 vs. 0.06 t ha^−1^), aboveground productivity (11.0 vs. 6.5 t ha^−1^ year^−1^), and litter production (7.3 vs. 6.1 t ha^−1^ year^−1^). In contrast, fine root biomass (3.7 vs. 5.1 t ha^−1^) and SOM (80.3 vs. 73.2 t ha^−1^) were not significantly different between wet and dry forests (Appendix S4: Table S2, Figure S1). On average, 70% of total biomass was stored in the upper 15 cm of the soil (wet forest [WF] = 68, dry forest [DF] = 74%), followed by 25% in aboveground living biomass (WF = 29, DF = 21%), 4.3% in fine root biomass (WF = 3.5, DF = 5.2%), and less than 0.5% in aboveground dead biomass (Table 1).

**TABLE 1:** The average percentage of aboveground living biomass, aboveground dead biomass, fine root biomass, and soil organic matter relative to total biomass (i.e., the sum of these components), along with their SE, in all forest types, dry forests, and wet forests.

Forest type and response variable	Average	SE
All forests		
Aboveground living biomass	25.2	1.5
Aboveground dead biomass	0.2	0.07
Fine root biomass	4.3	0.4
Soil organic matter	70.3	1.5
Wet forests		
Aboveground living biomass	28.7	2.2
Aboveground dead biomass	0.3	0.1
Fine root biomass	3.5	0.5
Soil organic matter	67.5	2.5
Dry forests		
Aboveground living biomass	21.3	1.4
Aboveground dead biomass	0.06	0.01
Fine root biomass	5.2	0.5
Soil organic matter	73.5	1.3

**FIGURE 2:**
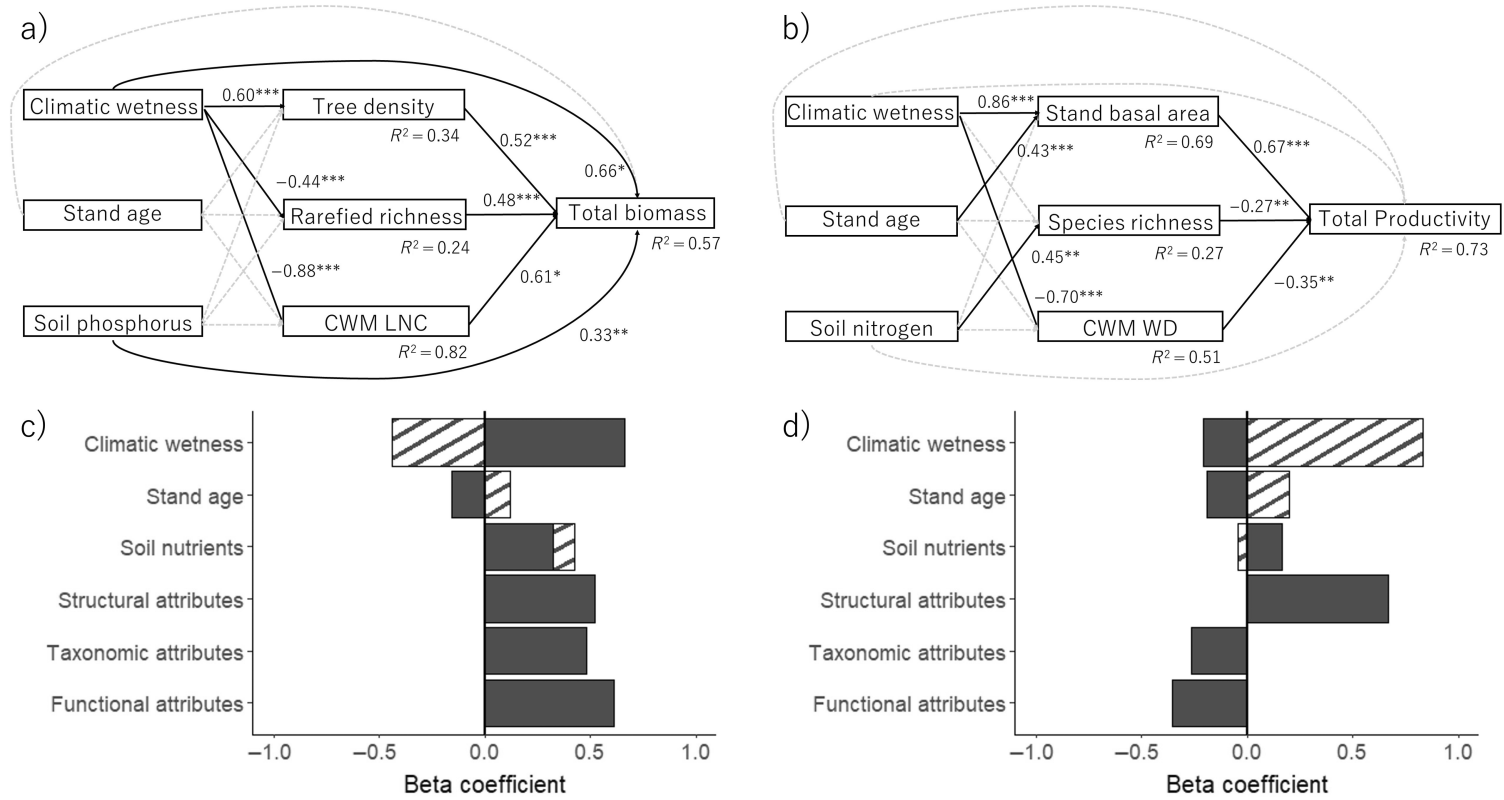
Structural equation models (SEM) for (a) total biomass stock (the sum of aboveground living biomass, aboveground dead biomass, fine root biomass, and soil organic matter, in tons per hectare); (b) total aboveground biomass productivity (the sum of aboveground biomass productivity and annual litter production, in tons per hectare per year); and bar graphs showing beta coefficients of each factor on (c) total biomass stock and (d) total aboveground biomass productivity based on (a) and (b). Direct and indirect effects of environmental conditions (climatic wetness, stand age, and soil nutrients) and direct effects of structural attributes (i.e., tree density or stand basal area), taxonomic attributes (i.e., species richness per plot or rarefied species richness per 150 stems), and functional attributes (i.e., a community‐weighted mean [CWM] leaf nitrogen concentration [LNC] or wood density [WD]) were evaluated. In (a) and (b), for all significant relations (continuous black arrows), the beta coefficient and significance level are given (*p < 0.05, **p < 0.01, ***p < 0.001), and for all nonsignificant relations (gray, dashed arrows), no statistics are shown. *R* ^2^ values show the explained variance of the response variables. In (c) and (d), the filled bars show the direct effects of environmental conditions and forest attributes, and the hatched bars show the indirect effects of environmental conditions. For more statistics on the structural equation models, see Appendix S4: Table S3.

**FIGURE 3:**
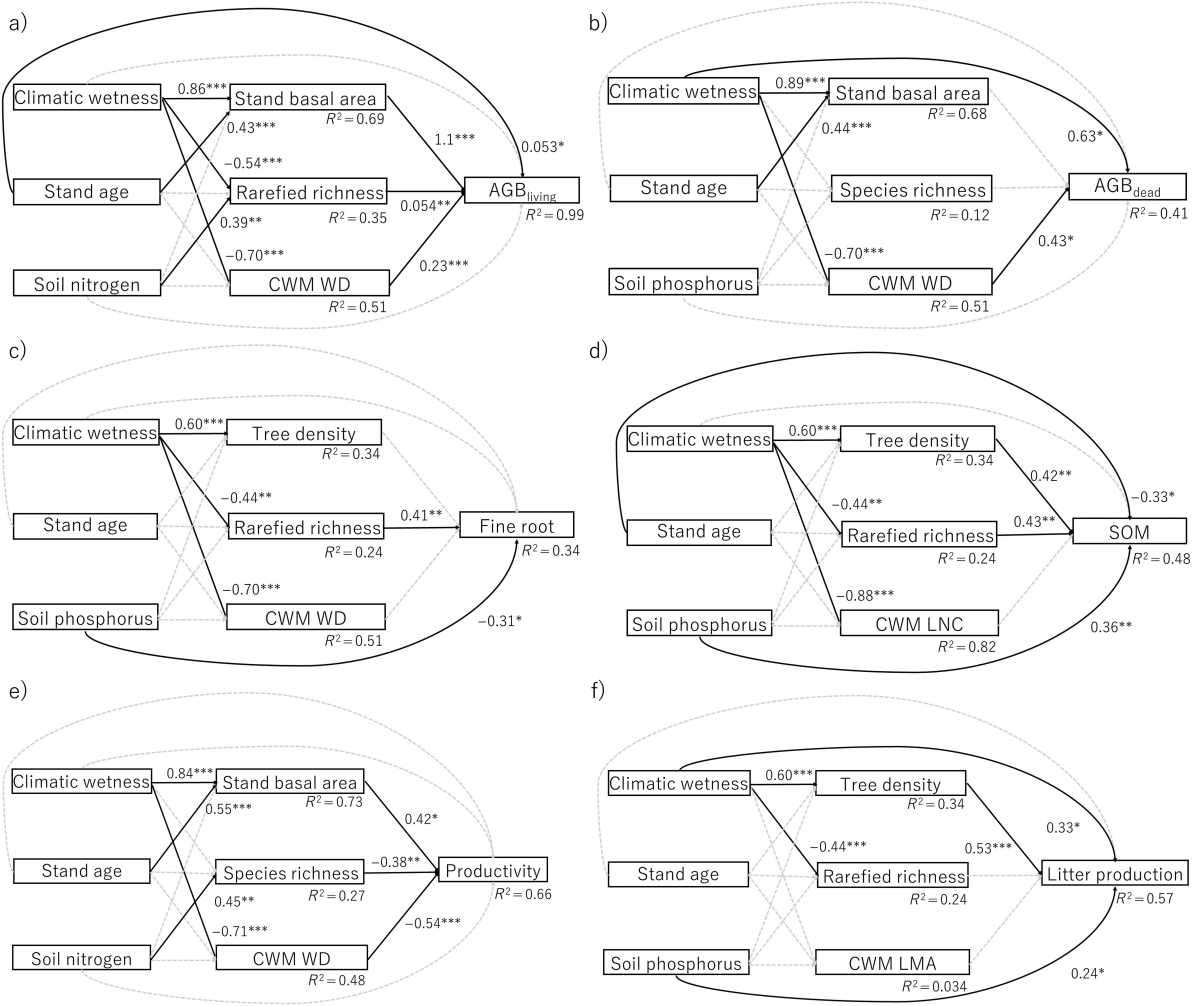
Structural equation models for (a) aboveground living biomass (AGB_living_, in tons per hectare), (b) aboveground dead biomass (AGB_dead_, in tons per hectare), (c) fine root biomass in the top 15 cm of the soil (fine root, in tons per hectare), (d) soil organic matter in the top 15 cm of the soil (SOM, in tons per hectare), (e) aboveground biomass productivity (productivity, in tons per hectare per year), and (f) litter production (in tons per hectare per year). Direct and indirect effects of environmental conditions (climatic wetness, stand age, and soil nutrients) and direct effects of structural attributes (i.e., tree density or stand basal area), taxonomic attributes (i.e., species richness per plot or rarefied species richness per 150 stems), and functional attributes (i.e., a community‐weighted mean [CWM] leaf nitrogen concentration [LNC], leaf mass per area [LMA], or wood density [WD]) were evaluated. For all significant relations (continuous black arrows), the beta coefficient and significance level are given (*p < 0.05, **p < 0.01, ***p < 0.001), and for all nonsignificant relations (gray, dashed arrows), no statistics are shown. *R* ^2^ values show the explained variance of the response variables. For more statistics on the structural equation models, see Appendix S4: Table S3.

To evaluate our conceptual model (Figure 1), we developed one SEM for total biomass stock (Figure 2a), one for total AGB productivity (Figure 2b), and one for each of the six compartments of biomass pools and productivity (Figure 3).

The best models explained, on average, 57% of the variation in response variables, ranging from 34% for fine root biomass to 99% for AGB_living_ (Appendix S4: Table S3). Wet forests (i.e., “climatic wetness” effect) exhibited significantly higher values than the dry forest for stand basal area and tree density but lower values for rarefied species richness, CWM WD, and CWM LNC (Figure 3). Stand basal area was higher in older forest stands, while other structural, taxonomic, and functional attributes did not vary significantly with the small range of stand age (2.3–3.6 years) considered in this study. Both species richness and rarefied species richness increased with soil N, but none of the forest attributes was significantly affected by soil P.

Total biomass was positively affected by all forest attributes (tree density, rarefied richness, CWM LNC) and two environmental conditions (climatic wetness and soil P) (Figure 2a,c). Total biomass productivity was mostly driven by forest attributes and increased with stand basal area and decreased with species richness and CWM WD (Figure 2b,d).

**FIGURE 4:**
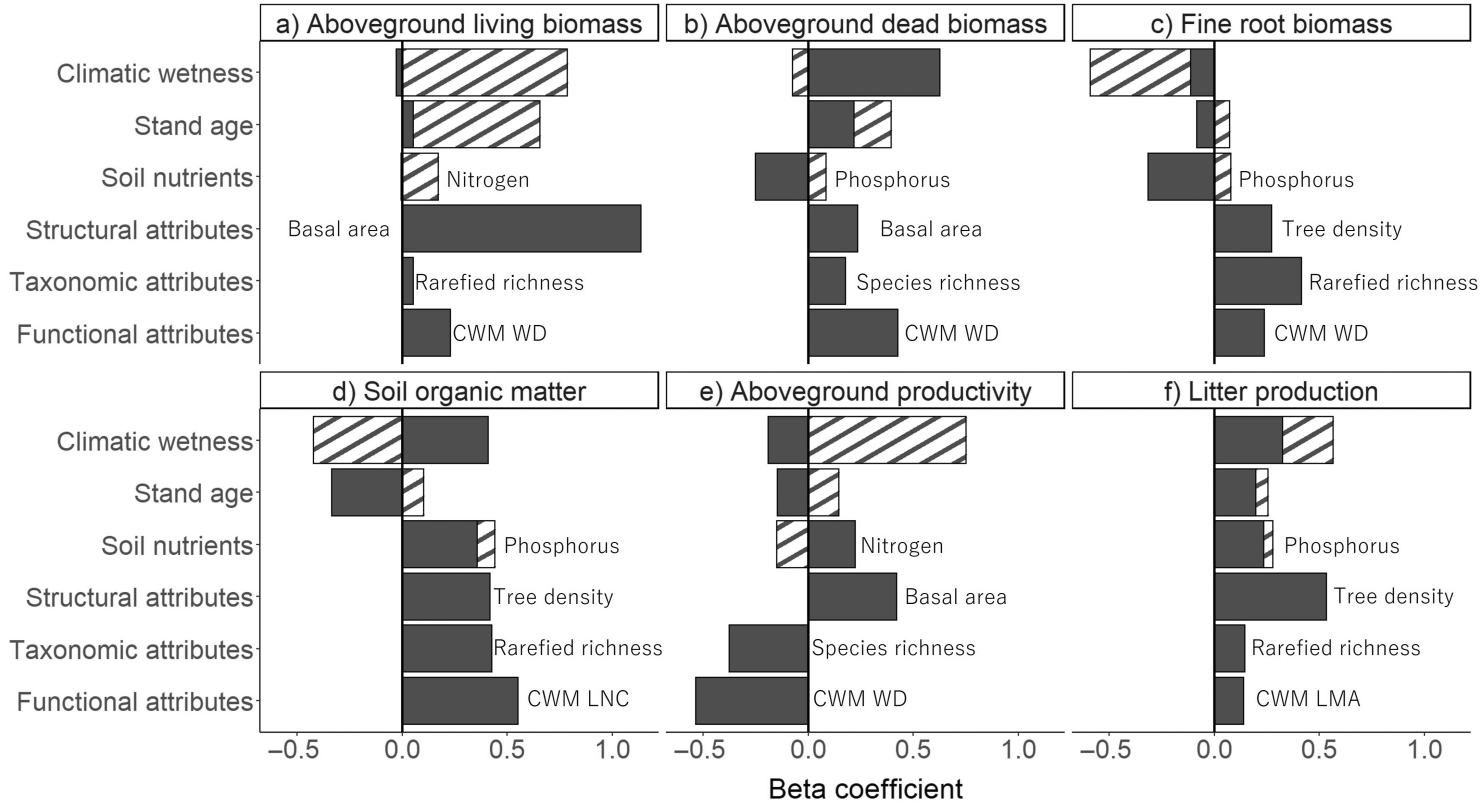
Beta coefficients of environmental conditions (climatic wetness, stand age and soil nutrients) and forest attributes (structural, taxonomic and functional attributes) on six compartments of biomass pools and productivity: (a) Aboveground living biomass (in tons per hectare), (b) aboveground dead biomass (in tons per hectare), (c) fine root biomass in the top 15 cm of the soil (in tons per hectare), (d) soil organic matter in the top 15 cm of the soil (in tons per hectare), (e) aboveground biomass productivity (in tons per hectare per year), and (f) litter production (in tons per hectare per year) based on the best models in structural equation models (Figure 3). The filled bars show the direct effects of environmental conditions and forest attributes, and the hatched bars show the indirect effects of environmental conditions.

**FIGURE 5:**
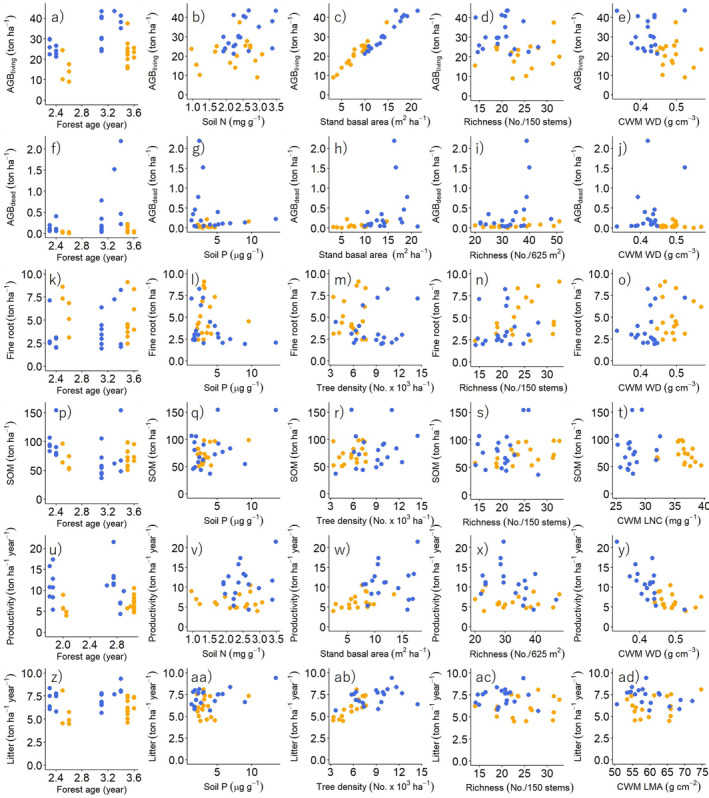
Bivariate relationships of stand age, one soil nutrient (i.e., soil nitrogen [N] or phosphorus [P]), one structural attribute (i.e., stand basal area or tree density), one taxonomic attribute (i.e., species richness per plot or rarefied species richness per 150 stems), and one functional attribute (community‐weighted mean [CWM] leaf nitrogen concentration [LNC], leaf mass per area [LMA], or wood density [WD]) compared with aboveground living biomass (ABG_living_, in tons per hectare), aboveground dead biomass (ABG_dead_, in tons per hectare), fine root biomass in the top 15 cm of the soil (fine root, in tons per hectare), soil organic matter in the top 15 cm of the soil (SOM, in tons per hectare), aboveground biomass productivity (productivity, in tons per hectare per year), and litter production (litter, in tons per hectare per year) in tropical dry (orange) and wet (blue) forests. The chosen environmental variables and forest attributes were the ones that were selected in the best model in the structural equation models (Figure 3). Note that these bivariate relationships are for illustration purposes only and may not necessarily provide the same results as in the structural equation models.

AGB_living_ was positively affected mainly by forest attributes (stand basal area, rarefied richness, CWM WD) and stand age. AGB_dead_ increased with climatic wetness and CWM WD (Figure 3a,b). Litter production and SOM both increased with soil P and forest structure (tree density). In addition, litter production increased with climatic wetness, while SOM increased with rarefied richness but decreased with stand age (Figure 3d,f). Fine root biomass increased with diversity (rarefied richness) and soil P, whereas aboveground productivity was only driven by forest attributes, increased with structure (stand basal area), and decreased with species richness and CWM WD (Figure 3c,e). Beta coefficients of direct and indirect effects of environmental conditions and forest attributes based on the best models of SEMs are summarized in Figure 4, and bivariate scatterplots for all relationships used in the SEMs are shown in Figure 5.

## DISCUSSION

We evaluated how environmental conditions and forest attributes determine productivity and biomass pools in different above‐ and belowground compartments at the onset of succession in dry and wet forests. The main findings are that: (1) the majority of ecosystem forest biomass was stored in the soil (70%) and to a lesser extent in the aboveground vegetation (25%); (2) environmental conditions and forest attributes similarly contributed to total biomass stocks, as total biomass stocks increased with climatic wetness, soil fertility (phosphorus), structural attributes (tree density), species diversity (rarefied richness), and functional trait composition (CWM LNC); and (3) climatic wetness and structural attributes (stand basal area) strongly increased total AGB productivity. Below, we discuss the underlying mechanisms and implications for tropical forest restoration and climate change mitigation.

### Forest attributes are most strongly affected by macroclimate

Overall, climatic wetness was most often significant and the strongest driver of all forest attributes. Stand age increased only stand basal area and soil N increased only species richness (Figure 3).

#### Structural attributes

As hypothesized, we found that stand basal area and tree density were higher in wet forests (Figure 3) because of more productive environmental conditions (cf. Rozendaal et al., 2017). Although the variation in stand age among plots is small (less than 1.5 years), stand basal area still increased with stand age (Figure 3a,b,e), reflecting the fast pace of tree growth and forest stand development in early successional tropical forests.

#### Taxonomic attributes

We hypothesized that species richness was larger in the wet than in the dry forest because of a larger regional species pool, but found that richness per plot did not differ significantly between the forest types, and rarefied species richness was even lower in wet forests than in dry forests (Figure 3). Rarefied richness generally increases with species evenness, because even abundances increase the chance of randomly selecting more species (Appendix S4: Table S1). Because wet forests had lower evenness (Appendix S4: Figure S1) due to the strong dominance of fast‐growing species and a longer tail of rare species (Rozendaal et al., 2019; van der Sande et al., 2024), this may lead to a lower rarefied richness in wet forests. Soil nutrients had surprisingly little effect on forest structural and functional attributes (Figure 3). Soil N only increased species richness and rarefied richness likely because low soil N indicates strong depletion during intense previous land use. Consequently, such severely degraded land may limit the number of species that can regenerate (Jakovac et al., 2016).

#### Functional attributes

We hypothesized that the wet forest would have a higher abundance of species with acquisitive trait values (e.g., high LNC and low WD) than the dry forest because of a more productive environment. Partly in line with our hypothesis, we found that both community WD and LNC were higher in dry forests (Figure 3), probably reflecting species adaptations to drought. High WD is associated with high cavitation resistance and therefore allows continued stem water transport during drought (Markesteijn et al., 2011; Pineda‐García et al., 2013). High LNC increases Rubisco concentration, which draws down CO_2_ concentration inside the leaves. This allows plants to increase photosynthetic water use efficiency by reducing their stomatal aperture while maintaining similar assimilation rates (Onoda et al., 2017; Querejeta et al., 2022).

### Environmental conditions and forest attributes determine biomass pools and productivity

We hypothesized and found that all environmental conditions and forest attributes affected biomass productivity and pools in different compartments, but the direction and strength of their effects varied among compartments (Figures 3 and 4). For environmental conditions, climatic wetness and soil phosphorus emerged as the most important drivers. For forest attributes, stand basal area, tree density, rarefied richness, and community WD emerged as the most important drivers.

#### AGB and productivity

AGB_living_ and productivity were mostly driven by forest attributes, whereas AGB_dead_ was driven by both environmental conditions and forest attributes (Figure 3a,b,e). AGB_living_ increased with stand basal area because larger trees store more biomass (Poorter, van der Sande, et al., 2015), and also increased with community WD because of increased biomass per unit stem volume (Finegan et al., 2015). AGB_living_ additionally increased weakly with species richness (Figure 3a), which could indicate that higher diversity leads to more efficient resource use complementarity and a greater chance to include a highly productive species (Loreau, 1998; Tilman, 1999). This ultimately should lead to a larger standing biomass. Yet, in our case, productivity declined with species richness probably because young tropical forests are dominated by a few fast‐growing pioneer species that contribute the most to productivity (Lohbeck et al., 2016). Hence, more diverse plots may contain more shade‐tolerant species with a slower growth rate, leading to lower plot‐level productivity but higher plot‐level biomass residence time through insurance effects, and thus biomass stocks (Loreau et al., 2021). AGB_living_ also increased weakly with stand age probably because of accumulated height growth over time (Matsuo, Bongers, et al., 2024).

AGB productivity increased with stand basal area (Figure 3e) because larger trees accumulate biomass faster than smaller trees (Stephenson et al., 2014). Productivity declined with community WD probably because dense‐wooded species tend to have narrower vessels and pit pores, and therefore a lower hydraulic conductivity, leaf stomatal conductance, and associated photosynthetic carbon gain (Santiago et al., 2004). Yet, these dense‐wooded species have higher survival rates (Poorter et al., 2010) and thus may still positively contribute to AGB_living_ (Figure 3a).

AGB_dead_ was higher in wet forests possibly because of the increased risk of biophysical hazards such as falling branches, herbivory, and pathogens, which increases tree mortality (Coley & Barone, 1996; Spear et al., 2015). AGB_dead_ was higher in forest stands with high community WD, probably because dense wood is more resistant to wood decay, resulting in a longer residence time of dead stems, and thus an accumulation of standing and lying deadwood in the forest (Chave et al., 2009; Yang et al., 2022).

#### Fine root biomass

Fine root biomass decreased with soil P probably because on fertile soils plants need to allocate less biomass to fine roots to acquire soil nutrients for their growth (Figure 3c) (Maycock & Congdon, 2000; Wurzburger & Wright, 2015). Fine root biomass increased with rarefied richness perhaps because of more efficient packing of the soil volume by roots (Brassard et al., 2013), although it should be noted that fine root sampling was only conducted in the top layer (0–15 cm), and thus complementary root packing is limited. Alternatively, low species richness is related to low soil N, which indicates the intense previous land use (Hordijk et al., 2024). These intense previous land‐use practices may not only deplete soil nitrogen but also alter other soil characteristics, such as soil structure and compaction, as well as soil microbial community, which ultimately reduces fine root biomass (Correa et al., 2019). Although we found these significant effects on fine root biomass, the explained variation of fine root biomass was the lowest (*R* ^2^ = 0.34) among all compartments (average *R* ^2^ = 0.57). To improve our understanding of factors driving fine root biomass stocks, future studies could include as predictors community root traits, such as fine root diameter, root tissue density, and specific root length (Bardgett et al., 2014; Zeng et al., 2020).

#### Litter production and SOM

Litter production and SOM are driven by similar factors; they increased with soil P and tree density (Figure 3d,f). On fertile soils and in wetter climates, litter production is greater likely because of (1) higher overall productivity (Figure 5e) (van der Sande, Arets, et al., 2017), and thus leaf and branch production rates; and (2) stronger competition for light than for nutrients, resulting in higher biomass allocation to stem, branches, and leaves (Chakravarty et al., 2019). Similarly, litter production increased with tree density probably because of (a) efficient spatial crown packing among different‐sized individuals (Hardiman et al., 2011), and (b) abundant smaller individuals that allocate proportionally more biomass to their leaves than large individuals (Poorter, Jagodzinski, et al., 2015). Increased litter production may, in turn, lead to higher SOM (Appendix S4: Figure S2) (Feng et al., 2019; Giweta, 2020). Furthermore, soils with high P concentration may exhibit faster decomposition rates of deadwood and litter, accompanied by increased biopedturbation from soil fauna, resulting in a faster carbon transfer to the soil (Prescott & Vesterdal, 2021). It is important to note that a fertility‐driven increase in metabolic activity of decomposer communities may also increase carbon release into the atmosphere through elevated respiration rates, thus reducing SOM (Curiel Yuste et al., 2007). SOM additionally increased with species richness perhaps because a larger diversity of species increases the chance of including a species with slowly decomposable litter. This in turn increases the recalcitrant organic carbon and favors more microorganisms, thereby facilitating carbon transfer (Freschet et al., 2012; García‐Palacios et al., 2016). Stand age had, independently from forest attributes, a negative effect on SOM, perhaps because previously cultivated cassava and corn have high productivity and turnover of leaves and fine roots, facilitating the rapid carbon accumulation in the soil during the active agricultural land use (Oldfield et al., 2019). After land abandonment, these organic materials further decompose and are released into the atmosphere through respiration. This leads to a reduction in SOM, though this is partly compensated for by the SOM derived from superficial tree roots and litter fall from regenerating forests.

#### The importance of environmental conditions and forest attributes

While climatic wetness exhibits limited direct effects on biomass pools and productivity, its overall contribution is substantial through indirect effects by shaping various forest attributes (Figures 3 and 4). In contrast, soil nutrients, especially soil P, have little effect on forest attributes but exhibit the most significant direct effect on biomass pools and productivity (Figures 3 and 4), highlighting the importance of the independent role of soil nutrients on ecosystem functioning. Besides commonly considered structural attributes, such as maximum tree diameter and stand basal area (Ali et al., 2019), we found that tree density plays a pivotal role, especially in litter production and SOM. This emphasizes the importance of smaller individuals and efficient crown packing for biomass turnover and accumulation in the soil in early successional forests. Rarefied species richness affected more response variables than species richness per plot (Figure 3), indicating that not only the number of species but also their even distribution of abundance affects ecosystem functioning (Hordijk et al., 2023). This is because species with different traits can contribute more to ecosystem functioning when they have a similar abundance (Lohbeck et al., 2016). Lastly, our stem trait (WD) affected more response variables than leaf traits (Figure 3), reflecting the fact that in forests the majority of AGB is stored in stems rather than in leaves (Poorter, Jagodzinski, et al., 2015).

### Implications for forest restoration

Growing evidence underscores the potential of natural regeneration as a low‐cost strategy to achieve ecosystem‐level carbon accumulation and stocks for global strategies and initiatives, such as ecosystem restoration (https://www.decadeonrestoration.org), land restoration (https://www.unccd.int/land‐and‐life/drought/toolbox/land‐restoration), climate change mitigation projects (https://www.bonnchallenge.org), and climate neutrality (https://climate.ec.europa.eu/eu‐action/climate‐strategies‐targets/2050‐long‐term‐strategy_en). Our study demonstrates that overall, tropical secondary forests rapidly recover and accumulate carbon in different compartments of the vegetation and soil, yet recovery rates could vary due to coarse‐ and fine‐scale variations in climatic and edaphic conditions. Based on the results of this study, implementing natural regeneration is especially recommended in (1) wet forests due to high AGB productivity through rapid structural development, which results in high AGB stocks; and (2) on fertile soils because high soil P can directly increase SOM by enhancing litter production and decomposition.

## CONCLUSIONS

Macroclimate strongly shapes forest attributes, which in turn determine biomass pools and productivity. Soil nutrients, especially soil P, strongly drive ecosystem functioning (in three out of the six evaluated cases). This supports the notion that soil P is the most limiting soil nutrient for tropical ecosystems growing on strongly weathered soils (van der Sande, Arets, et al., 2017; Vitousek et al., 2010). Structural attributes, such as stand basal area and tree density, strongly enhance different compartments of biomass pools and productivity. Species diversity and a more conservative trait composition increase forest biomass pools, while they decrease productivity, suggesting that both quantity and the identity of species determine ecosystem functioning. Future research can further explore these relationships to gain a holistic understanding of how ecosystem functioning recovers during forest succession along climatic and edaphic gradients.

## AUTHOR CONTRIBUTIONS

Tomonari Matsuo, Lourens Poorter, Masha T. van der Sande, and Lucy Amissah planned and designed the research. Tomonari Matsuo and Lucy Amissah led the fieldwork, and Salim Mohammed Abdul, Dieudonne Wedaga Koyiba, Justice Opoku, Bas de Wit, and Tijs Kuzee contributed to the data collection. Tomonari Matsuo performed data analysis. Lourens Poorter and Masha T. van der Sande contributed to data analysis. Lucy Amissah, Bas de Wit, and Tijs Kuzee provided comments. Tomonari Matsuo, Lourens Poorter, Masha T. van der Sande, and Lucy Amissah wrote the manuscript. Salim Mohammed Abdul, Dieudonne Wedaga Koyiba, Justice Opoku, Bas de Wit, and Tijs Kuzee provided comments. All the authors contributed critically to the drafts and gave final approval for publication.

## FUNDING INFORMATION

Tomonari Matsuo, Lourens Poorter, and Masha T. van der Sande were supported by the European Research Council Advanced Grant PANTROP 834775. Tomonari Matsuo was also supported by the Shikata Memorial Trust for Nature Conservation, Japan. Masha T. van der Sande was also supported by the Veni research program NWO‐VI.Veni.192.027.

## CONFLICT OF INTEREST STATEMENT

The authors declare no conflicts of interest.

## Supporting information


Appendix S1:

Appendix S2:

Appendix S3:

Appendix S4:

## Data Availability

Data (Matsuo, Poorter, et al., 2024) are available in DANS (Data Archiving and Networked Services) at https://doi.org/10.17026/LS/FRDEFQ.

## References

[ecy4488-bib-0001] Addo‐Fordjour, P. , and Z. B. Rahmad . 2013. “Mixed Species Allometric Models for Estimating Above‐Ground Liana Biomass in Tropical Primary and Secondary Forests, Ghana.” International Scholalry Research Notices 2013: 1–9.

[ecy4488-bib-0002] Aghimien, E. V. , B. Osikabor , M. S. Adedeji , and O. T. Adams . 2020. “Volume Techniques for Estimating Standing and Lying Dead Wood in Okomu National Park, Edo State, Nigeria.” Biometrics & Biostatistics International Journal 9: 111–116.

[ecy4488-bib-0003] Ali, A. , S. L. Lin , J. K. He , F. M. Kong , J. H. Yu , and H. S. Jiang . 2019. “Big‐Sized Trees Overrule Remaining Trees' Attributes and Species Richness as Determinants of Aboveground Biomass in Tropical Forests.” Global Change Biology 25: 2810–2824. 31120573 10.1111/gcb.14707

[ecy4488-bib-0004] Amissah, L. , G. M. J. Mohren , B. Kyereh , V. K. Agyeman , and L. Poorter . 2018. “Rainfall Seasonality and Drought Performance Shape the Distribution of Tropical Tree Species in Ghana.” Ecology and Evolution 8: 8582–8597. 30250725 10.1002/ece3.4384 PMC6144999

[ecy4488-bib-0005] Bardgett, R. D. , L. Mommer , and F. T. De Vries . 2014. “Going Underground: Root Traits as Drivers of Ecosystem Processes.” Trends in Ecology & Evolution 29: 692–699. 25459399 10.1016/j.tree.2014.10.006

[ecy4488-bib-0006] Beer, C. , M. Reichstein , E. Tomelleri , P. Ciais , M. Jung , N. Carvalhais , C. Rödenbeck , et al. 2010. “Covariation with Climate.” Science 329: 834–839. 20603496 10.1126/science.1184984

[ecy4488-bib-0007] Bonan, G. B. 2008. “Forests and Climate Change: Forcings, Feedbacks, and the Climate Benefits of Forests.” Science 320: 1444–1449. 18556546 10.1126/science.1155121

[ecy4488-bib-0008] Brassard, B. W. , H. Y. H. Chen , X. Cavard , J. Laganière , P. B. Reich , Y. Bergeron , D. Paré , and Z. Yuan . 2013. “Tree Species Diversity Increases Fine Root Productivity through Increased Soil Volume Filling.” Journal of Ecology 101: 210–219.

[ecy4488-bib-0009] Bray, R. H. , and L. T. Kurtz . 1945. “Determination of Total, Organic, and Available Forms of Phosphorus in Soils.” Soil Science 59: 39–46.

[ecy4488-bib-0010] Bremner, J. M. , and D. R. Keeney . 1965. “Steam Distillation Methods for Determination of Ammonium, Nitrate and Nitrite.” Analytica Chimica Acta 32: 485–495.

[ecy4488-bib-0011] Camenzind, T. , S. Hättenschwiler , K. K. Treseder , A. Lehmann , and M. C. Rillig . 2018. “Nutrient Limitation of Soil Microbial Processes in Tropical Forests.” Ecological Monographs 88: 4–21.

[ecy4488-bib-0012] Chakravarty, S. , P. Rai , N. A. Pala , and G. Shukla . 2019. “Litter Production and Decomposition in Tropical Forest.” In Handbook of Research on the Conservation and Restoration of Tropical Dry Forests, edited by R. Bhadouria , S. Tripathi , P. Srivastava , and P. Singh , 193–212. Pennsylvania, USA: IGI Global.

[ecy4488-bib-0013] Chao, A. , and C. H. Chiu . 2016. “Species Richness: Estimation and Comparison.” Wiley StatsRef: Statistics Reference Online 1: 26.

[ecy4488-bib-0014] Chao, A. , N. J. Gotelli , T. C. Hsieh , E. L. Sander , K. H. Ma , R. K. Colwell , and A. M. Ellison . 2014. “Rarefaction and Extrapolation with Hill Numbers: A Framework for Sampling and Estimation in Species Diversity Studies.” Ecological Monographs 84: 45–67.

[ecy4488-bib-0015] Chave, J. , D. Coomes , S. Jansen , S. L. Lewis , N. G. Swenson , and A. E. Zanne . 2009. “Towards a Worldwide Wood Economics Spectrum.” Ecology Letters 12: 351–366. 19243406 10.1111/j.1461-0248.2009.01285.x

[ecy4488-bib-0016] Chazdon, R. L. , S. G. Letcher , M. van Breugel , M. Martínez‐Ramos , F. Bongers , and B. Finegan . 2007. “Rates of Change in Tree Communities of Secondary Neotropical Forests Following Major Disturbances.” Philosophical Transactions of the Royal Society of London, Series B – Biological Sciences 362: 273–289. 17255036 10.1098/rstb.2006.1990 PMC2311434

[ecy4488-bib-0017] Coley, P. D. , and J. A. Barone . 1996. “Herbivory and Plant Defenses in Tropical Forests.” Annual Review of Ecology and Systematics 27: 305–335.

[ecy4488-bib-0018] Correa, J. , J. A. Postma , M. Watt , and T. Wojciechowski . 2019. “Soil Compaction and the Architectural Plasticity of Root Systems.” Journal of Experimental Botany 70: 6019–6034. 31504740 10.1093/jxb/erz383 PMC6859514

[ecy4488-bib-0019] Curiel Yuste, J. , D. D. Baldocchi , A. Gershenson , A. Goldstein , L. Misson , and S. Wong . 2007. “Microbial Soil Respiration and Its Dependency on Carbon Inputs, Soil Temperature and Moisture.” Global Change Biology 13: 2018–2035.

[ecy4488-bib-0020] Djagbletey, G. D. , S. Adu‐Bredu , A. DUah‐Gyamfi , R. Aabeyir , E. D. Djagbletey , S. E. Akpalu , G. K. Adeyiga , et al. 2020. “Wood Density Handbook for Some West African Trees.” In FAO and CSIR. Kumasi, Ghana: University Printing Press, Kwame Nkrumah University of Science and Technology.

[ecy4488-bib-0021] Ellsworth, D. S. , and P. B. Reich . 1996. “Photosynthesis and Leaf Nitrogen in Five Amazonian Tree Species during Early Secondary Succession.” Ecology 77: 581–594.

[ecy4488-bib-0022] Enriquez, S. , C. M. Duarte , and K. Sand‐Jensen . 1993. “Patterns in Decomposition Rates among Photosynthetic Organisms: The Importance of Detritus C:N:P Content.” Oecologia 94: 457–471. 28313985 10.1007/BF00566960

[ecy4488-bib-0023] Evans, J. R. 1989. “Photosynthesis and Nitrogen Relationships in Leaves of C3 Plants.” Oecologia 78: 9–19. 28311896 10.1007/BF00377192

[ecy4488-bib-0024] Feng, C. , Z. Wang , Y. Ma , S. Fu , and H. Y. H. Chen . 2019. “Increased Litterfall Contributes to Carbon and Nitrogen Accumulation Following Cessation of Anthropogenic Disturbances in Degraded Forests.” Forest Ecology and Management 432: 832–839.

[ecy4488-bib-0025] Finegan, B. , M. Peña‐Claros , A. de Oliveira , N. Ascarrunz , M. S. Bret‐Harte , G. Carreño‐Rocabado , F. Casanoves , et al. 2015. “Does Functional Trait Diversity Predict Above‐Ground Biomass and Productivity of Tropical Forests? Testing Three Alternative Hypotheses.” Journal of Ecology 103: 191–201.

[ecy4488-bib-0026] Forestry Division . 1963. Afram Headwaters Group Forest Reserve Plan. Accra, Ghana: Forestry Department, the Government of Ghana.

[ecy4488-bib-0027] Freschet, G. T. , R. Aerts , and J. H. C. Cornelissen . 2012. “A Plant Economics Spectrum of Litter Decomposability.” Functional Ecology 26: 56–65.

[ecy4488-bib-0028] Freschet, G. T. , L. Pagès , C. M. Iversen , L. H. Comas , B. Rewald , C. Roumet , J. Klimešová , et al. 2021. “A Starting Guide to Root Ecology: Strengthening Ecological Concepts and Standardising Root Classification, Sampling, Processing and Trait Measurements.” New Phytologist 232: 973–1122. 34608637 10.1111/nph.17572 PMC8518129

[ecy4488-bib-0029] García‐Palacios, P. , E. A. Shaw , D. H. Wall , and S. Hättenschwiler . 2016. “Temporal Dynamics of Biotic and Abiotic Drivers of Litter Decomposition.” Ecology Letters 19: 554–563. 26947573 10.1111/ele.12590

[ecy4488-bib-0030] Giweta, M. 2020. “Role of Litter Production and Its Decomposition, and Factors Affecting the Processes in a Tropical Forest Ecosystem: A Review.” Journal of Ecology and Environment 44: 1–9.

[ecy4488-bib-0031] Grime, J. P. 1998. “Benefits of Plant Diversity to Ecosystems: Immediate, Filter and Founder Effects.” Journal of Ecology 86: 902–910.

[ecy4488-bib-0032] Hall, J. B. , and M. D. Swaine . 1981. Distribution and Ecology of Vascular Plants in a Tropical Rain Forest Forest Vegetation in Ghana. The Hague: Dr W. Junk Publishers.

[ecy4488-bib-0033] Hardiman, B. S. , G. Bohrer , C. M. Gough , C. S. Vogel , and P. S. Curtis . 2011. “The Role of Canopy Structural Complexity in Wood Net Primary Production of a Maturing Northern Deciduous Forest.” Ecology 92: 1818–1827. 21939078 10.1890/10-2192.1

[ecy4488-bib-0034] Help, C. H. R. , P. M. J. Herman , and K. Soetaert . 1998. “Indices of Diversity and Evenness.” Oceanis 24: 61–88.

[ecy4488-bib-0035] Hordijk, I. , D. S. Maynard , S. P. Hart , M. Lidong , H. ter Steege , J. Liang , S. de‐Miguel , et al. 2023. “Evenness Mediates the Global Relationship between Forest Productivity and Richness.” Journal of Ecology 111: 1308–1326.

[ecy4488-bib-0036] Hordijk, I. , L. Poorter , M. Martínez‐Ramos , F. Bongers , R. D. L. Mendoza , P. J. Romero , M. van der Sande , et al. 2024. “Land Use Legacies Affect Early Tropical Forest Succession in Mexico.” Applied Vegetation Science 27: e12784.

[ecy4488-bib-0037] Hossain, M. A. , A. R. Anik , N. Chakma , K. Johnson , M. Henry , R. Jalal , O. Carrillo , et al. 2019. Estimation Procedures of Indicators and Variables of the Bangladesh Forest Inventory. Dhaka: Forest Department and Food and Agriculture Organization of the United Nations.

[ecy4488-bib-0038] IPBES . 2019. Global Assessment Report on Biodiversity and Ecosystem Services of the Intergovernmental Science‐Policy Platform on Biodiversity and Ecosystem Services. Edited by E. S. Brondizio , J. Settele , S. Díaz , and H. T. Ngo . Bonn: IPBES Secretariat.

[ecy4488-bib-0039] Jakovac, C. C. , F. Bongers , T. W. Kuyper , R. C. G. Mesquita , and M. Peña‐Claros . 2016. “Land Use as a Filter for Species Composition in Amazonian Secondary Forests.” Journal of Vegetation Science 27: 1104–1116.

[ecy4488-bib-0040] Jones, I. L. , S. J. DeWalt , O. R. Lopez , L. Bunnefeld , Z. Pattison , and D. H. Dent . 2019. “Above‐ and Belowground Carbon Stocks Are Decoupled in Secondary Tropical Forests and Are Positively Related to Forest Age and Soil Nutrients Respectively.” Science of the Total Environment 697: 133987. 31484096 10.1016/j.scitotenv.2019.133987

[ecy4488-bib-0041] Keenan, R. J. , G. A. Reams , F. Achard , J. V. de Freitas , A. Grainger , and E. Lindquist . 2015. “Dynamics of Global Forest Area: Results from the FAO Global Forest Resources Assessment 2015.” Forest Ecology and Management 352: 9–20.

[ecy4488-bib-0042] Kenzo, T. , T. Ichie , D. Hattori , T. Itioka , C. Handa , T. Ohkubo , J. J. Kendawang , et al. 2009. “Development of Allometric Relationships for Accurate Estimation of Above‐ and Below‐Ground Biomass in Tropical Secondary Forests in Sarawak, Malaysia.” Journal of Tropical Ecology 25: 371–386.

[ecy4488-bib-0043] Kitajima, K. , and L. Poorter . 2010. “Tissue‐Level Leaf Toughness, but Not Lamina Thickness, Predicts Sapling Leaf Lifespan and Shade Tolerance of Tropical Tree Species.” New Phytologist 186: 708–721. 20298481 10.1111/j.1469-8137.2010.03212.x

[ecy4488-bib-0044] Kramer‐Walter, K. R. , P. J. Bellingham , T. R. Millar , R. D. Smissen , S. J. Richardson , and D. C. Laughlin . 2016. “Root Traits Are Multidimensional: Specific Root Length Is Independent from Root Tissue Density and the Plant Economic Spectrum.” Journal of Ecology 104: 1299–1310.

[ecy4488-bib-0045] Lehnebach, R. , R. Beyer , V. Letort , and P. Heuret . 2018. “The Pipe Model Theory Half a Century On: A Review.” Annals of Botany 121: 773–795. 29370362 10.1093/aob/mcx194 PMC5906905

[ecy4488-bib-0046] Lei, P. , M. Scherer‐Lorenzen , and J. Bauhus . 2012. “The Effect of Tree Species Diversity on Fine‐Root Production in a Young Temperate Forest.” Oecologia 169: 1105–1115. 22298110 10.1007/s00442-012-2259-2

[ecy4488-bib-0047] Lohbeck, M. , F. Bongers , M. Martinez‐Ramos , and L. Poorter . 2016. “The Importance of Biodiversity and Dominance for Multiple Ecosystem Functions in a Human‐Modifed Tropical Landscape.” Ecology 97: 2772–2779. 27859119 10.1002/ecy.1499

[ecy4488-bib-0048] Lohbeck, M. , L. Poorter , M. Martínez‐ramos , and F. Bongers . 2015. “Biomass Is the Main Driver of Changes in Ecosystem Process Rates during Tropical Forest Succession.” Ecology 96: 1242–1252. 26236838 10.1890/14-0472.1

[ecy4488-bib-0049] Loreau, M. 1998. “Separating Sampling and Other Effects in Biodiversity Experiments.” Oikos 82: 600–602.

[ecy4488-bib-0050] Loreau, M. , M. Barbier , E. Filotas , D. Gravel , F. Isbell , S. J. Miller , J. M. Montoya , et al. 2021. “Biodiversity as Insurance: From Concept to Measurement and Application.” Biological Reviews 96: 2333–2354. 34080283 10.1111/brv.12756 PMC8519139

[ecy4488-bib-0051] Malhi, Y. , L. E. O. C. Aragão , D. B. Metcalfe , R. Paiva , C. A. Quesada , S. Almeida , L. Anderson , et al. 2009. “Comprehensive Assessment of Carbon Productivity, Allocation and Storage in Three Amazonian Forests.” Global Change Biology 15: 1255–1274.

[ecy4488-bib-0052] Markesteijn, L. , L. Poorter , H. Paz , L. Sack , and F. Bongers . 2011. “Ecological Differentiation in Xylem Cavitation Resistance Is Associated with Stem and Leaf Structural Traits.” Plant, Cell and Environment 34: 137–148. 10.1111/j.1365-3040.2010.02231.x 20946587

[ecy4488-bib-0053] Martin, P. A. , A. C. Newton , and J. M. Bullock . 2013. “Carbon Pools Recover More Quickly than Plant Biodiversity in Tropical Secondary Forests.” Proceedings of the Royal Society B: Biological Sciences 280: 20132236. 10.1098/rspb.2013.2236 PMC3826225 24197410

[ecy4488-bib-0054] Matsuo, T. , L. Amissah , J. Kok , and L. Poorter . 2023. “Regeneración natural de bosques tropicales en campos agrícolas abandonados en Ghana.” Boletín de la SCME 3: 44–53.

[ecy4488-bib-0055] Matsuo, T. , F. Bongers , M. Martínez‐Ramos , M. T. van der Sande , and L. Poorter . 2024. “Height Growth and Biomass Partitioning during Secondary Succession Differ among Forest Light Strata and Successional Guilds in a Tropical Rainforest.” Oikos 6: e10486.

[ecy4488-bib-0056] Matsuo, T. , T. Hiura , and Y. Onoda . 2022. “Vertical and Horizontal Light Heterogeneity along Gradients of Secondary Succession in Cool and Warm Temperate Forests.” Journal of Vegetation Science 33: e13135. 37274931 10.1111/jvs.13135 PMC10234446

[ecy4488-bib-0057] Matsuo, T. , M. Martínez‐Ramos , F. Bongers , M. T. van der Sande , and L. Poorter . 2021. “Forest Structure Drives Changes in Light Heterogeneity during Tropical Secondary Forest Succession.” Journal of Ecology 109: 2871–2884. 34588706 10.1111/1365-2745.13680 PMC8453511

[ecy4488-bib-0058] Matsuo, T. , M. Martínez‐Ramos , Y. Onoda , F. Bongers , M. Lohbeck , and L. Poorter . 2024. “Light Competition Drives Species Replacement during Secondary Tropical Forest Succession.” Oecologia 205: 1–11. 38727828 10.1007/s00442-024-05551-w PMC11144147

[ecy4488-bib-0059] Matsuo, T. , L. Poorter , M. T. van der Sande , S. M. Abdul , D. W. Koyiba , J. Opoku , B. de Wit , T. Kuzee , and L. Amissah . 2024. “Replication Data for ‘Drivers of Biomass Stocks and Productivity of Tropical Secondary Forests’.” DANS Data Station Life Sciences. 10.17026/LS/FRDEFQ. PMC11737357 39629674

[ecy4488-bib-0060] Matsuo, T. , M. T. van der Sande , L. Amissah , J. Dabo , S. Mohammed Abdul , and L. Poorter . 2024. “Herbaceous Species and Dry Forest Species Have More Acquisitive Leaf Traits than Woody Species and Wet Forest Species.” Functional Ecology 38: 194–205.

[ecy4488-bib-0061] Maycock, C. R. , and R. A. Congdon . 2000. “Fine Root Biomass and Soil N and P in North Queensland Rain Forests.” Biotropica 32: 185–190.

[ecy4488-bib-0062] Meiners, S. J. , S. T. Pickett , and M. L. Cadenasso . 2015. An Integrative Approach to Successional Dynamics. Cambridge, UK: Cambridge University Press.

[ecy4488-bib-0063] Morriën, E. , S. E. Hannula , L. B. Snoek , N. R. Helmsing , H. Zweers , M. De Hollander , R. L. Soto , et al. 2017. “Soil Networks Become More Connected and Take Up More Carbon as Nature Restoration Progresses.” Nature Communications 8: 14349. 10.1038/ncomms14349 PMC5309817 28176768

[ecy4488-bib-0064] Nelson, D. A. , and L. Sommers . 1983. “Total Carbon, Organic Carbon, and Organic Matter.” Methods of Soil Analysis: Part 2 Chemical and Microbiological Properties 9: 539–579.

[ecy4488-bib-0065] Neumann, M. , S. Echeverria , and H. Hasenauer . 2023. “A Simple Concept for Estimating Deadwood Carbon in Forests.” Carbon Management 14: 2197762.

[ecy4488-bib-0066] Odum, E. P. 1969. “The Strategy of Ecosystem Development.” Science 164: 262–270. 5776636 10.1126/science.164.3877.262

[ecy4488-bib-0067] Oldfield, E. E. , M. A. Bradford , and S. A. Wood . 2019. “Global Meta‐Analysis of the Relationship between Soil Organic Matter and Crop Yields.” The Soil 5: 15–32.

[ecy4488-bib-0068] Olsen, S. R. , and L. E. Sommers . 1982. “Part 2‐Chemical and Microbiological Properties.” In Methods of Soil Analysis, edited by A. L. Page , 403–430. Madison, WI: American Society of Agronomy.

[ecy4488-bib-0069] Onoda, Y. , I. J. Wright , J. R. Evans , K. Hikosaka , K. Kitajima , Ü. Niinemets , H. Poorter , T. Tosens , and M. Westoby . 2017. “Physiological and Structural Tradeoffs Underlying the Leaf Economics Spectrum.” New Phytologist 214: 1447–1463. 28295374 10.1111/nph.14496

[ecy4488-bib-0070] Pan, N. , S. Wang , F. Wei , M. Shen , and B. Fu . 2021. “Inconsistent Changes in NPP and LAI Determined from the Parabolic LAI versus NPP Relationship.” Ecological Indicators 131: 108134.

[ecy4488-bib-0071] Pérez‐Harguindeguy, N. , S. Díaz , E. Garnier , S. Lavorel , H. Poorter , P. Jaureguiberry , M. S. Bret‐Harte , et al. 2013. “New Handbook for Standardised Measurement of Plant Functional Traits Worldwide.” Australian Journal of Botany 61: 167–234.

[ecy4488-bib-0072] Pineda‐García, F. , H. Paz , and F. C. Meinzer . 2013. “Drought Resistance in Early and Late Secondary Successional Species from a Tropical Dry Forest: The Interplay between Xylem Resistance to Embolism, Sapwood Water Storage and Leaf Shedding.” Plant, Cell and Environment 36: 405–418. 10.1111/j.1365-3040.2012.02582.x 22812458

[ecy4488-bib-0073] Poorter, H. , A. M. Jagodzinski , R. Ruiz‐peinado , S. Kuyah , Y. Luo , J. Oleksyn , V. A. Usoltsev , T. N. Buckley , P. B. Reich , and L. Sack . 2015. “How Does Biomass Distribution Change with Size and Differ among Species? An Analysis for 1200 Plant Species from Five Continents.” New Phytologist 208: 736–749. 26197869 10.1111/nph.13571 PMC5034769

[ecy4488-bib-0074] Poorter, H. , Ü. Niinemets , N. Ntagkas , A. Siebenkäs , M. Mäenpää , S. Matsubara , and T. L. Pons . 2019. “A Meta‐Analysis of Plant Responses to Light Intensity for 70 Traits Ranging from Molecules to Whole Plant Performance.” New Phytologist 223: 1073–1105. 30802971 10.1111/nph.15754

[ecy4488-bib-0075] Poorter, L. , F. Bongers , T. M. Aide , A. M. A. Zambrano , P. Balvanera , J. M. Becknell , V. Boukili , et al. 2016. “Biomass Resilience of Neotropical Secondary Forests.” Nature 530: 211–214. 26840632 10.1038/nature16512

[ecy4488-bib-0076] Poorter, L. , I. McDonald , A. Alarcón , E. Fichtler , J. C. Licona , M. Peña‐Claros , F. Sterck , Z. Villegas , and U. Sass‐Klaassen . 2010. “The Importance of Wood Traits and Hydraulic Conductance for the Performance and Life History Strategies of 42 Rainforest Tree Species.” New Phytologist 185: 481–492. 19925555 10.1111/j.1469-8137.2009.03092.x

[ecy4488-bib-0077] Poorter, L. , M. T. van der Sande , E. J. M. M. Arets , N. Ascarrunz , B. Enquist , B. Finegan , J. C. Licona , et al. 2017. “Biodiversity and Climate Determine the Functioning of Neotropical Forests.” Global Ecology and Biogeography 26: 1423–1434.

[ecy4488-bib-0078] Poorter, L. , M. T. van der Sande , J. Thompson , E. J. M. M. Arets , A. Alarcón , J. Álvarez‐Sánchez , N. Ascarrunz , et al. 2015. “Diversity Enhances Carbon Storage in Tropical Forests.” Global Ecology and Biogeography 24: 1314–1328.

[ecy4488-bib-0079] Prescott, C. E. , and L. Vesterdal . 2021. “Decomposition and Transformations along the Continuum from Litter to Soil Organic Matter in Forest Soils.” Forest Ecology and Management 498: 119522.

[ecy4488-bib-0080] Puletti, N. , R. Canullo , W. Mattioli , R. Gawryś , P. Corona , and J. Czerepko . 2019. “A Dataset of Forest Volume Deadwood Estimates for Europe.” Annals of Forest Science 76: 1–8.

[ecy4488-bib-0081] Querejeta, J. I. , I. Prieto , C. Armas , F. Casanoves , J. S. Diémé , M. Diouf , H. Yossi , B. Kaya , F. I. Pugnaire , and G. M. Rusch . 2022. “Higher Leaf Nitrogen Content Is Linked to Tighter Stomatal Regulation of Transpiration and More Efficient Water Use across Dryland Trees.” New Phytologist 235: 1351–1364. 35582952 10.1111/nph.18254 PMC9542767

[ecy4488-bib-0082] Reich, P. B. 2014. “The World‐Wide ‘Fast‐Slow’ Plant Economics Spectrum: A Traits Manifesto.” Journal of Ecology 102: 275–301.

[ecy4488-bib-0083] Rosseel, Y. 2012. “Lavaan: An R Package for Structural Equation Modeling.” Journal of Statistical Software 48: 1–36.

[ecy4488-bib-0084] Rozendaal, D. M. A. , F. Bongers , T. M. Aide , E. Alvarez‐dávila , N. Ascarrunz , P. Balvanera , J. M. Becknell , et al. 2019. “Biodiversity Recovery of Neotropical Secondary Forests.” Science Advances 5: 1–10. 10.1126/sciadv.aau3114 PMC6402850 30854424

[ecy4488-bib-0085] Rozendaal, D. M. A. , R. L. Chazdon , F. Arreola‐Villa , P. Balvanera , T. V. Bentos , J. M. Dupuy , J. L. Hernández‐Stefanoni , et al. 2017. “Demographic Drivers of Aboveground Biomass Dynamics during Secondary Succession in Neotropical Dry and Wet Forests.” Ecosystems 20: 340–353.

[ecy4488-bib-0086] Santiago, L. S. , G. Goldstein , F. C. Meinzer , J. B. Fisher , K. Machado , D. Woodruff , and T. Jones . 2004. “Leaf Photosynthetic Traits Scale with Hydraulic Conductivity and Wood Density in Panamanian Forest Canopy Trees.” Oecologia 140: 543–550. 15232729 10.1007/s00442-004-1624-1

[ecy4488-bib-0087] Shinozaki, K. , K. Yoda , K. Hozumi , and T. Kira . 1964. “A Quantitative Analysis of Plant Form – The Pipe Model Theory I. Basic Analysis.” Japanese Journal of Ecology 14: 97–105.

[ecy4488-bib-0088] Sierra Cornejo, N. , D. Hertel , J. N. Becker , A. Hemp , and C. Leuschner . 2020. “Biomass, Morphology, and Dynamics of the Fine Root System across a 3,000‐M Elevation Gradient on Mt. Kilimanjaro.” Frontiers in Plant Science 11: 13. 32117363 10.3389/fpls.2020.00013 PMC7010809

[ecy4488-bib-0089] Spear, E. R. , P. D. Coley , and T. A. Kursar . 2015. “Do Pathogens Limit the Distributions of Tropical Trees across a Rainfall Gradient?” Journal of Ecology 103: 165–174.

[ecy4488-bib-0090] Stephenson, N. L. , A. J. Das , R. Condit , S. E. Russo , P. J. Baker , N. G. Beckman , D. A. Coomes , et al. 2014. “Rate of Tree Carbon Accumulation Increases Continuously with Tree Size.” Nature 507: 90–93. 24429523 10.1038/nature12914

[ecy4488-bib-0091] Teixeira, H. M. , I. M. Cardoso , F. J. J. A. Bianchi , A. da Cruz Silva , D. Jamme , and M. Peña‐Claros . 2020. “Linking Vegetation and Soil Functions during Secondary Forest Succession in the Atlantic Forest.” Forest Ecology and Management 457: 117696.

[ecy4488-bib-0092] Terzaghi, M. , A. Montagnoli , A. Di Iorio , G. S. Scippa , and D. Chiatante . 2013. “Fine‐Root Carbon and Nitrogen Concentration of European Beech (*Fagus sylvatica* L.) in Italy Prealps: Possible Implications of Coppice Conversion to High Forest.” Frontiers in Plant Science 4: 192. 23785374 10.3389/fpls.2013.00192 PMC3680728

[ecy4488-bib-0093] Tilman, D. 1999. “The Ecological Consequences of Changes in Biodiversity: A Search for General Principles.” Ecology 80: 1455–1474.

[ecy4488-bib-0094] van Breugel, M. , D. Craven , H. R. Lai , M. Baillon , B. L. Turner , and J. S. Hall . 2019. “Soil Nutrients and Dispersal Limitation Shape Compositional Variation in Secondary Tropical Forests across Multiple Scales.” Journal of Ecology 107: 566–581.

[ecy4488-bib-0095] van der Sande, M. T. , E. J. M. M. Arets , M. Peña‐Claros , M. R. Hoosbeek , Y. Cáceres‐Siani , P. van der Hout , and L. Poorter . 2017. “Soil Fertility and Species Traits, but Not Diversity, Drive Productivity and Biomass Stocks in a Guyanese Tropical Rainforest.” Functional Ecology 32: 461–474.

[ecy4488-bib-0096] van der Sande, M. T. , M. Peña‐Claros , N. Ascarrunz , E. J. M. M. Arets , J. C. Licona , M. Toledo , and L. Poorter . 2017. “Abiotic and Biotic Drivers of Biomass Change in a Neotropical Forest.” Journal of Ecology 105: 1223–1234.

[ecy4488-bib-0097] van der Sande, M. T. , L. Poorter , G. Derroire , M. M. do Espirito Santo , M. Lohbeck , S. C. Müller , R. Bhaskar , et al. 2024. “Tropical Forest Succession Increases Tree Taxonomic and Functional Tree Richness but Decreases Evenness.” Global Ecology and Biogeography e13856.

[ecy4488-bib-0098] van der Sande, M. T. , J. S. Powers , T. W. Kuyper , N. Norden , B. Salgado‐Negret , J. Silva De Almeida , F. Bongers , et al. 2022. “Soil Resistance and Recovery during Neotropical Forest Succession.” Philosophical Transactions of the Royal Society B: Biological Sciences 378: 20210074. 10.1098/rstb.2021.0074 PMC9661943 36373919

[ecy4488-bib-0099] Violle, C. , M.‐L. Navas , D. Vile , E. Kazakou , C. Fortunel , I. Hummel , and E. Garnier . 2007. “Let the Concept of Trait be Functional!” Oikos 116: 882–892.

[ecy4488-bib-0100] Vitousek, P. M. , S. Porder , B. Z. Houlton , and O. A. Chadwick . 2010. “Terrestrial Phosphorus Limitation: Mechanisms, Implications, and Nitrogen‐Phosphorus Interactions.” Ecological Applications 20: 5–15. 20349827 10.1890/08-0127.1

[ecy4488-bib-0101] Westoby, M. , and I. J. Wright . 2006. “Land‐Plant Ecology on the Basis of Functional Traits.” Trends in Ecology & Evolution 21: 261–268. 16697912 10.1016/j.tree.2006.02.004

[ecy4488-bib-0102] Wright, I. J. , P. B. Reich , M. Westoby , D. D. Ackerly , Z. Baruch , F. Bongers , J. Cavender‐Bares , et al. 2004. “The Worldwide Leaf Economics Spectrum.” Nature 428: 821–827. 15103368 10.1038/nature02403

[ecy4488-bib-0103] Wurzburger, N. , and S. J. Wright . 2015. “Fine‐Root Responses to Fertilization Reveal Multiple Nutrient Limitation in a Lowland Tropical Forest.” Ecology 96: 2137–2146. 26405739 10.1890/14-1362.1

[ecy4488-bib-0104] Yang, S. , L. Poorter , E. E. Kuramae , U. Sass‐Klaassen , M. F. A. Leite , O. Y. A. Costa , G. A. Kowalchuk , et al. 2022. “Stem Traits, Compartments and Tree Species Affect Fungal Communities on Decaying Wood.” Environmental Microbiology 24: 3625–3639. 35229433 10.1111/1462-2920.15953 PMC9544286

[ecy4488-bib-0105] Yuan, Z. , A. Ali , T. Jucker , P. Ruiz‐Benito , S. Wang , L. Jiang , X. Wang , et al. 2019. “Multiple Abiotic and Biotic Pathways Shape Biomass Demographic Processes in Temperate Forests.” Ecology 100: 1–10. 10.1002/ecy.2650 PMC6849813 30742311

[ecy4488-bib-0106] Yuan, Z. , A. Ali , S. Wang , A. Gazol , R. Freckleton , X. Wang , F. Lin , et al. 2018. “Abiotic and Biotic Determinants of Coarse Woody Productivity in Temperate Mixed Forests.” Science of the Total Environment 630: 422–431. 29482149 10.1016/j.scitotenv.2018.02.125

[ecy4488-bib-0107] Zeng, W. , W. Xiang , J. Fang , B. Zhou , S. Ouyang , Y. Zeng , L. Chen , P. Lei , A. Milcu , and O. J. Valverde‐Barrantes . 2020. “Species Richness and Functional‐Trait Effects on Fine Root Biomass along a Subtropical Tree Diversity Gradient.” Plant and Soil 446: 515–527.

