# Supplementary material for: Drivers of biomass stocks and productivity of tropical secondary forests
Source: Ecology. 2024 Dec 4;106(1):e4488. doi: 10.1002/ecy.4488 (PMC11737357; doi:10.1002/ecy.4488)
Supplement: Supplementary file 1 — Appendix S1: [file ECY-106-e4488-s001.pdf]

Drivers of biomass stocks and productivity of tropical secondary forests

Tomonari Matsuo, Lourens Poorter, Masha T. van der Sande, Salim Mohammed Abdul, Dieudonne Wedaga Koyiba, Justice Opoku, Bas de Wit, Tijs Kuzee, Lucy Amissah

Journal: Ecology

Appendix S1: Detailed methods for developing a new allometric equation for estimating aboveground biomass of shrub and tree species in Ghana.

In total, 144 trees and 9 shrubs representing 16 species, 14 genera, and 8 families were harvested in dry forests in 2023, and 191 trees and 40 shrubs representing 25 species, 22 genera, and 17 families were harvested in wet forests in 2022 and measured for aboveground parts. Individuals with damaged crowns or broken trunks were not considered. Harvested trees were on average 4.6 cm (range 0.74-26.0) in diameter at breast height (DBH, 1.3 m) and 6.3 m (1.5-23.1) in height in dry forests, and 2.8 cm (0.47-15.0) in DBH and 4.5 m (1.3-16.5) in height in wet forests. The size range of the DBH and height, and all selected species were typical in both study sites. After harvesting, samples were divided into leaves, branches, and main stems in the field. The total fresh weight of each part was measured in the field and then representative samples were dried in the laboratory of CSIR-FORIG (Forestry Research Institute of Ghana) in Kumasi to determine moisture content (Kenzo et al., 2009). These samples were oven-dried at 80°C for 72 hours until they reached constant mass. DBH and wood density (WD, g cm^{-3}) were used as independent variables to develop the allometric equation for tree and shrub species.

References

Kenzo, T., Furutani, R., Hattori, D., Kendawang, J. J., Tanaka, S., Sakurai, K., & Ninomiya, I. (2009). Allometric equations for accurate estimation of above-ground biomass in logged-over tropical rainforests in Sarawak, Malaysia. *Journal of Forest Research*, *14*(6), 365–372. https://doi.org/10.1007/s10310-009-0149-1
