# Supplementary material for: Drivers of biomass stocks and productivity of tropical secondary forests
Source: Ecology. 2024 Dec 4;106(1):e4488. doi: 10.1002/ecy.4488 (PMC11737357; doi:10.1002/ecy.4488)
Supplement: Supplementary file 2 — Appendix S2: [file ECY-106-e4488-s003.pdf]

Drivers of biomass stocks and productivity of tropical secondary forests

Tomonari Matsuo, Lourens Poorter, Masha T. van der Sande, Salim Mohammed Abdul, Dieudonne Wedaga Koyiba, Justice Opoku, Bas de Wit, Tijs Kuzee, Lucy Amissah

Journal: Ecology

Appendix S2: Collection and calculation of fine root biomass and soil organic matter.
A soil ring with a 5 cm diameter was used to collect the fine roots to a depth of 15 cm (i.e., total volume: 295 cm^3). A simple soil ring was chosen as the method given its ease of use and on average low impact on the sampling plots (Freschet et al., 2021). Because in many places rocks were present below 15 cm, sampling was limited to this depth. Eight samples were taken for each plot to capture spatial heterogeneity. Root samples were properly washed after soaking them in water for up to 24 hours (Freschet et al., 2021), and sieved with a 0.25 mm mesh sieve. Root samples were then oven-dried at 65°C for 48 hours and weighed. Samples were separated into fine (<2mm diameter) and coarse (>2mm) roots, which were weighed separately on a scale (units: grams). We only used the data of fine roots for the analysis.

For soil organic matter (SOM, $g\ g^{-1}$), using the same soil ring, four soil samples were collected per plot at depths of 0-15 cm. The samples were then brought to CSIR-SRI (Soil Research Institute) in Kumasi, where they were analyzed for SOM using the modified dichromate oxidation method of Walkley-Black (Nelson & Sommers, 1983). SOM was then converted to $g\ cm^{-3}$ by multiplying the mean bulk density ($g\ cm^{-3}$) of each plot. These values of root biomass and SOM per plot were scaled to ton ha^{-1} of 15 cm depth (1500 m^3), to compare values with aboveground living and dead biomass storage, which are also expressed in ton ha^{-1}.

References

Freschet, G. T., Pagès, L., Iversen, C. M., Comas, L. H., Rewald, B., Roumet, C., Klimešová, J., Zadworny, M., Poorter, H., Postma, J. A., Adams, T. S., Bagniewska-Zadworna, A., Bengough, A. G., Blancaflor, E. B., Brunner, I., Cornelissen, J. H. C., Garnier, E., Gessler, A., Hobbie, S. E., … McCormack, M. L. (2021). A starting guide to root ecology: strengthening ecological concepts and standardising root classification, sampling, processing and trait measurements. *New Phytologist*, *232*(3), 973–1122. https://doi.org/10.1111/nph.17572

Nelson, D. A., & Sommers, L. (1983). Total carbon, organic carbon, and organic matter. *Methods of Soil Analysis: Part 2 Chemical and Microbiological Properties*, *9*, 539–579.
