# Supplementary material for: Drivers of biomass stocks and productivity of tropical secondary forests
Source: Ecology. 2024 Dec 4;106(1):e4488. doi: 10.1002/ecy.4488 (PMC11737357; doi:10.1002/ecy.4488)
Supplement: Supplementary file 3 — Appendix S3: [file ECY-106-e4488-s002.pdf]

Drivers of biomass stocks and productivity of tropical secondary forests

Tomonari Matsuo, Lourens Poorter, Masha T. van der Sande, Salim Mohammed Abdul, Dieudonne Wedaga Koyiba, Justice Opoku, Bas de Wit, Tijs Kuzee, Lucy Amissah

Journal: Ecology

Appendix S3: Detailed methods on leaf and stem trait measurements.

For each species, leaf traits were measured for two sunlit leaves for four to five young woody plants. The height of the sampled woody plants ranged between 1 and 8 m, and their stem diameter at 30 cm height ranged between 1 and 10 cm, which is a typical size range in early successional forests. Leaf samples were collected from outside of the permanent plots using machetes, tall pole pruners, and slingshots. The leaf collection was done during the wet season (between June and July in 2021 and April in 2022). After the collection, the weight of leaves was immediately measured in the field to determine leaf fresh weight (g). Leaves were scanned to estimate leaf area (LA, cm^2) using pixel counting software ImageJ (National Institutes of Health, Bethesda, MD, USA). The petiole was excluded from leaf trait measurements. The leaves were dried in the oven to constant weight (at 70 °C for 48 hours) and then weighed to determine leaf dry mass (g). Leaf mass per area (LMA, g cm^{-2}) was calculated as leaf dry mass divided by leaf area, and Leaf dry matter content (LDMC, %) as leaf dry mass divided by leaf fresh mass. Leaf nitrogen concentration (LNC, mg g^{-1}) and leaf phosphorus concentration (LPC, mg g^{-1}) were measured in the laboratory at Wageningen University & Research in the Netherlands. Oven-dried leaves were digested using a mixture of H_2SO_4–Se and salicylic acid (Novozamsky et al., 1983). The digestion process begins with H_2O_2, which oxidizes most of the organic matter. After decomposing the excess H_2O_2 and evaporating water, the digestion is completed with concentrated H_2SO_4 at an elevated temperature (330°C) using Se as a catalyst. Total N and P in the leaf digests were measured spectrophotometrically with a segmented-flow system (Skalar San++ System).

Wood density was based on wood cores (0.43 cm diameter), using an increment borer (Haglöf Sweden, Langsele, Sweden), and fresh volume was calculated with its diameter (0.43 cm) and length (L, cm) (Eq. S1)

$$\text{Fresh volume} = \pi \times (d/4)^2 \times L \qquad \text{(Eq. S1)}$$

Stem slices were sampled for species with small stems. For those samples, the fresh volume was determined with the water displacement method. Wood density (WD, g cm^{-3}) was calculated as oven-dried mass (at 80 ℃ for 48 hours) over fresh volume. This measurement was taken in the study area for 61 species studied; data on WD for the remaining species were taken from the wood density database in Ghana (Djagbletey et al., 2020). For each species, stem samples were collected from three adult individuals at the beginning of the wet season (March 2023). All traits were measured following standardized protocols (Pérez-Harguindeguy et al., 2013).

References

Djagbletey, G. D., Adu-Bredu, S., Duah-Gyamfi, A., Aabeyir, R., Djagbletey, E. D., Akpalu, S. E., Adeyiga, G. K., Addo-Danso, S. D., Hagan Brown, W., Dabo, J., & Amponsah-Manu, E. (2020). *Wood Density Handbook for some West African Trees*.

Novozamsky, I., Houba, V. J. G., van Eck, R., & van Vark, W. (1983). A novel digestion technique for multi-element plant analysis. *Communications in Soil Science and Plant Analysis*, *14*(3), 239–248. https://doi.org/10.1080/00103628309367359

Pérez-Harguindeguy, N., Díaz, S., Garnier, E., Lavorel, S., Poorter, H., Jaureguiberry, P., Bret-Harte, M. S., Cornwell, W. K., Craine, J. M., Gurvich, D. E., Urcelay, C., Veneklaas, E. J., Reich, P. B., Poorter, L., Wright, I. J., Ray, P., Enrico, L., Pausas, J. G., De Vos, A. C., … Cornelissen, J. H. C. (2013). New handbook for standardised measurement of plant functional traits worldwide. *Australian Journal of Botany*, *61*(3), 167–234. https://doi.org/10.1071/BT12225
