# Supplementary material for: Drivers of biomass stocks and productivity of tropical secondary forests
Source: Ecology. 2024 Dec 4;106(1):e4488. doi: 10.1002/ecy.4488 (PMC11737357; doi:10.1002/ecy.4488)
Supplement: Supplementary file 4 — Appendix S4: [file ECY-106-e4488-s004.pdf]

Drivers of biomass stocks and productivity of tropical secondary forests

Tomonari Matsuo, Lourens Poorter, Masha T. van der Sande, Salim Mohammed Abdul, Dieudonne Wedaga Koyiba, Justice Opoku, Bas de Wit, Tijs Kuzee, Lucy Amissah

Journal: Ecology

Appendix S4.

Table S1: Results of a multiple regression analysis with rarefied species richness per 150 stems as a response variable, and species richness per plot and species evenness based on Hill numbers (see methods for the detail) as predictor variables. SE indicates standard error.

Response variable	Predictor variable	Estimate	SE	t-value	p-value
Rarefied richness	Intercept	0.53	3.8	0.14	0.89
	Species richness	0.57	0.085	6.7	<0.001
	Species evenness	7.9	3.90	2.0	0.052

Table S2: All variables with abbreviation, description, what it indicates, units, mean, standard error, and ranges of values across plots for each forest type (dry or wet).

Abbreviation	Description	Indicator of	Units	Dry	Wet
Stand age	Age of the forest stand in 2023	Time since land abandonment	year	3.2 ± 0.1 (2.5-3.6)	2.8 ± 0.1 (2.3-3.4)
Soil pH	Soil pH	Soil toxicity		6.8 ± 0.1 (5.6-7.8)	4.9 ± 0.1 (4.1-5.6)
Soil N	Total nitrogen in soil	Soil fertility	mg g^{-1}	2.2 ± 0.2 (0.95-3.1)	2.5 ± 0.1 (1.9-3.5)
Soil P	Plant available phosphorus in soil	Soil fertility	μg g^{-1}	3.5 ± 0.4 (2.1-9.6)	4.1 ± 0.7 (1.2-13.5)
Soil K	Soil potassium content	Soil fertility	Cmol kg^{-1}	0.39 ± 0.05 (0.14-0.81)	0.15 ± 0.01 (0.086-0.32)
Sand	Soil sand content	Soil physical property	%	67.1 ± 3.1 (42.0-86.0)	43.2 ± 2.3 (30.0-76.0)
Silt	Soil silt content	Soil physical property	%	20.9 ± 1.7 (10.0-34.0)	35.6 ± 1.9 (14.0-46.0)
Clay	Soil clay content	Soil physical property	%	12.0 ± 1.9 (4.0-32.0)	21.2 ± 1.6 (6.0-34.0)
BD	Soil bulk density	Soil physical property	g cm^{-3}	1.2 ± 0.06 (0.68-1.6)	1.2 ± 0.08 (0.59-1.7)
Stand basal area	Sum of tree basal area per ha	Stand size	m^2 ha^{-1}	8.2 ± 0.7 (3.2-14.7)	14.4 ± 0.8 (9.9-21.3)
DBHmax	maximum tree size expressed as 95% quantile in DBH of trees	Large size tree	cm	8.8 ± 0.4 (6.5-12.2)	9.1 ± 0.5 (6.2-14.5)
Tree density	Number of trees per plot (625 m^2)	Stand density	#	363 ± 22.1 (208-477)	548 ± 38.3 (229-903)
Species richness	Number of species per plot	Niche complementarity	#	33.4 ± 2.2 (20.0-51.0)	32.8 ± 1.6 (22.0-48.0)
Rarefied richness	Rarefied species richness per 150 stems	Niche complementarity	#	24.6 ± 1.3 (14.0-32.8)	20.0 ± 0.88 (14.1-28.0)
Evenness	Species evenness based on Hill numbers	Niche complementarity		0.42 ± 0.05 (0.13-0.95)	0.29 ± 0.02 (0.17-0.44)
CWM LNC	Community weighted mean of leaf nitrogen concentration	Photosynthetic capacity	mg g^{-1}	36.4 ± 0.5 (32.0-39.4)	28.0 ± 0.5 (25.1-32.5)
CMW LPC	Community weighted mean of leaf phosphorus concentration	Growth capacity	mg g^{-1}	1.9 ± 0.02 (1.7-2.1)	1.4 ± 0.02 (1.2-1.6)
CWM LMA	Community weighted mean of leaf mass per area	Leaf decomposability and longevity	g cm^{-2}	60.9 ± 1.4 (53.5-74.6)	59.7 ± 1.3 (50.6-72.1)
CWM LDMC	Community weighted mean of leaf dry matter content	Leaf decomposability and longevity	%	33.2 ± 0.3 (30.8-34.7)	32.1 ± 0.8 (25.7-40.0)
CWM WD	Community weighted mean of wood density	Volume growth, wood defense	g cm^{-3}	0.49 ± 0.01 (0.45-0.57)	0.42 ± 0.01 (0.33-0.52)
AGB_{living}	Stem and crown biomass of living trees	Biomass storage	ton ha^{-1}	21.1 ± 1.7 (9.0-37.5)	31.2 ± 1.8 (21.2-43.6)
AGB_{dead}	Stem biomass of dead trees	Biomass storage	ton ha^{-1}	0.058 ± 0.01 (0.006-0.22)	0.37 ± 0.13 (0.036-2.2)
Fine root	Fine root biomass in top 15 cm of the soil	Biomass storage	ton ha^{-1}	5.1 ± 0.5 (2.4-9.1)	3.7 ± 0.5 (1.9-8.3)
SOM	Soil organic matter in top 15 cm of the soil	Biomass storage	ton ha^{-1}	73.2 ± 4.1 (50.7-98.8)	80.3 ± 7.6 (36.7-154.4)
Productivity	Annual biomass increment rate	Biomass productivity	ton ha^{-1} $year^{-1}$	6.5 ± 0.4 (4.0-10.5)	11.0 ± 1.0 (4.3-21.5)
Litter production	Annual leaf, branch, flower and seed production rate	Biomass productivity	ton ha^{-1} $year^{-1}$	6.1 ± 0.3 (4.5-8.1)	7.3 ± 0.2 (5.7-9.4)

Table S3: Results for the eight structural equation models (SEMs) of a) total biomass stock (the sum of aboveground living biomass, aboveground dead biomass, fine root biomass, and soil organic matter), b) total aboveground biomass productivity (the sum of aboveground biomass productivity and litter production), c) aboveground living biomass, d) aboveground dead biomass, e) fine root biomass, f) soil organic matter, g) aboveground biomass productivity and h) litter production (see also Fig. 2 and Fig. 3). The standardized regression coefficients (Std. coeff), Z-values and p-values are given for all regressions (i.e. all arrows in Fig. 2 and Fig. 3), and the R^2 of the endogenous variables (i.e. variables that are affected by other variables: forest attributes and forest biomass measures). All eight models were accepted (p = 0.053, 0.19, 0.26, 0.13, 0.30, 0.053, 0.15, 0.12; and $\chi 2$ = 7.7, 4.8, 4.0, 5.6, 3.7, 7.7, 5.4, 5.8, respectively). For abbreviations, see Appendix S4: Table S2.

**SEM response variable**	**SEM predictor variable**	**Std. coeff**	**Z- value**	**p- value**
a) Total biomass stock	Climatic wetness	0.66	2.2	0.028
	Stand age	-0.16	-1.2	0.24
	Soil P	0.33	2.7	0.008
	Tree density	0.52	3.6	<0.001
	Rarefied richness	0.48	3.5	<0.001
	CWM LNC	0.61	2.2	0.03
Tree density	Climatic wetness	0.60	4.0	<0.001
	Stand age	0.099	0.66	0.51
	Soil P	0.079	0.58	0.56
Rarefied richness	Climatic wetness	-0.44	-2.7	0.007
	Stand age	0.087	0.54	0.59
	Soil P	0.15	1.0	0.31
CWM LNC	Climatic wetness	-0.88	-11.3	<0.001
	Stand age	0.046	0.59	0.56
	Soil P	-0.024	-0.33	0.74
R^2 Total biomass storage	0.57			

R^2 Tree density	0.34			
R^2 Rarefied richness	0.24			
R^2 CWM LNC	0.82			
b) Total biomass productivity	Climatic wetness	-0.21	-1.1	0.26
	Stand age	-0.19	-1.6	0.11
	Soil N	0.17	1.6	0.11
	Stand basal area	0.67	4.3	<0.001
	Species richness	-0.27	-2.7	<0.008
	CWM WD	-0.35	-2.9	0.004
Stand basal area	Climatic wetness	0.86	8.1	<0.001
	Stand age	0.43	4.2	<0.001
	Soil N	0.13	1.3	0.19
Species richness	Climatic wetness	-0.048	-0.29	0.77
	Stand age	0.26	1.6	0.11
	Soil N	0.45	3.0	0.003
CWM WD	Climatic wetness	-0.70	-5.2	<0.001
	Stand age	0.050	0.39	0.70
	Soil N	0.028	-0.23	0.82
R^2 Total biomass productivity	0.73			
R^2 Stand basal area	0.69			
R^2 Species richness	0.27			
R^2 CWM WD	0.51			
c) Aboveground living biomass	Climatic wetness	-0.027	-0.75	0.45
	Stand age	0.053	2.4	0.016
	Soil N	-0.005	-0.24	0.81
	Stand basal area	1.1	39.0	<0.001

	Rarefied richness	0.054	2.7	0.008
	CWM WD	0.23	9.9	<0.001
Stand basal area	Climatic wetness	0.86	8.1	<0.001
	Stand age	0.43	4.2	<0.001
	Soil N	0.13	1.3	0.19
Rarefied richness	Climatic wetness	-0.54	-3.5	<0.001
	Stand age	0.044	0.30	0.77
	Soil N	0.39	2.8	0.006
CWM WD	Climatic wetness	-0.70	-5.2	<0.001
	Stand age	0.05	0.39	0.70
	Soil N	0.028	0.23	0.82
R^2 Aboveground living biomass	0.99			
R^2 Stand basal area	0.69			
R^2 Rarefied richness	0.35			
R^2 CWM WD	0.51			
d) Aboveground dead biomass	Climatic wetness	0.63	2.3	0.020
	Stand age	0.22	1.2	0.22
	Soil P	-0.25	-1.9	0.059
	Stand basal area	0.24	1.0	0.30
	Species richness	0.18	1.3	0.20
	CWM WD	0.43	2.3	0.025
Stand basal area	Climatic wetness	0.89	8.5	<0.001
	Stand age	0.44	4.2	<0.001
	Soil P	0.065	0.68	0.50
Species richness	Climatic wetness	0.072	0.41	0.68
	Stand age	0.30	1.8	0.078
	Soil P	0.18	1.1	0.26

CWM WD	Climatic wetness	-0.70	-5.4	<0.001
	Stand age	0.047	0.37	0.71
	Soil P	0.087	0.74	0.46
R^2 Aboveground dead biomass	0.41			
R^2 Stand basal area	0.68			
R^2 Species richness	0.12			
R^2 CWM WD	0.51			
e) Fine root biomass	Climatic wetness	-0.11	-0.47	0.64
	Stand age	-0.083	-0.55	0.59
	Soil P	-0.31	-2.2	0.027
	Tree density	0.27	1.6	0.11
	Rarefied richness	0.41	2.6	0.008
	CWM WD	0.24	1.2	0.23
Tree density	Climatic wetness	0.60	4.0	<0.001
	Stand age	0.099	0.66	0.51
	Soil P	0.079	0.58	0.56
Rarefied richness	Climatic wetness	-0.44	-2.7	0.007
	Stand age	0.087	0.54	0.59
	Soil P	0.15	1.0	0.31
CWM WD	Climatic wetness	-0.70	-5.4	<0.001
	Stand age	0.047	0.37	0.71
	Soil P	0.087	0.74	0.46
R^2 Fine root biomass	0.34			
R^2 Tree density	0.34			

R^2 Rarefied richness	0.24			
R^2 CWM WD	0.51			
f) Soil organic matter	Climatic wetness	0.41	1.3	0.21
	Stand age	-0.33	-2.3	0.02
	Soil P	0.36	2.7	0.007
	Tree density	0.42	2.6	0.008
	Rarefied richness	0.43	2.9	0.004
	CWM LNC	0.55	1.8	0.068
Tree density	Climatic wetness	0.60	4.0	<0.001
	Stand age	0.099	0.66	0.51
	Soil P	0.079	0.58	0.56
Rarefied richness	Climatic wetness	-0.44	-2.7	0.007
	Stand age	0.087	0.54	0.59
	Soil P	0.15	1.0	0.31
CWM LNC	Climatic wetness	-0.88	-11.3	<0.001
	Stand age	0.046	0.59	0.56
	Soil P	-0.024	-0.33	0.74
R^2 Soil organic matter	0.48			
R^2 Tree density	0.34			
R^2 Rarefied richness	0.24			
R^2 CWM LNC	0.82			
g) Aboveground productivity	Climatic wetness	-0.19	-0.88	0.38
	Stand age	-0.16	-1.0	0.30
	Soil N	0.21	1.8	0.072
	Stand basal area	0.44	2.3	0.023

	Species richness	-0.37	-3.2	0.001
	CWM WD	-0.54	-3.9	<0.001
Stand basal area	Climatic wetness	0.84	8.4	<0.001
	Stand age	0.55	5.7	<0.001
	Soil N	0.12	1.3	0.20
Species richness	Climatic wetness	-0.027	-0.16	0.87
	Stand age	0.24	1.5	0.13
	Soil N	0.44	2.9	0.004
CWM WD	Climatic wetness	-0.71	-5.1	<0.001
	Stand age	-0.014	-0.11	0.91
	Soil N	0.053	0.42	0.68
R^2 Aboveground productivity	0.65			
R^2 Stand basal area	0.73			
R^2 Species richness	0.26			
R^2 CWM WD	0.48			
h) Litter production	Climatic wetness	0.33	2.0	0.044
	Stand age	0.20	1.6	0.12
	Soil P	0.24	2.0	0.044
	Tree density	0.53	3.8	<0.001
	Rarefied richness	0.15	1.1	0.26
	CWM LMA	0.14	1.2	0.22
Tree density	Climatic wetness	0.60	4.0	<0.001
	Stand age	0.099	0.66	0.51
	Soil P	0.079	0.58	0.56
Rarefied richness	Climatic wetness	-0.44	-2.7	0.007
	Stand age	0.087	0.54	0.59
	Soil P	0.15	1.0	0.31

CWM LMA	Climatic wetness	-0.11	-0.62	0.54
	Stand age	-0.049	-0.27	0.79
	Soil P	-0.14	-0.85	0.40
R^2 Litter production	0.57			
R^2 Tree density	0.34			
R^2 Rarefied richness	0.24			
R^2 CWM LMA	0.034			

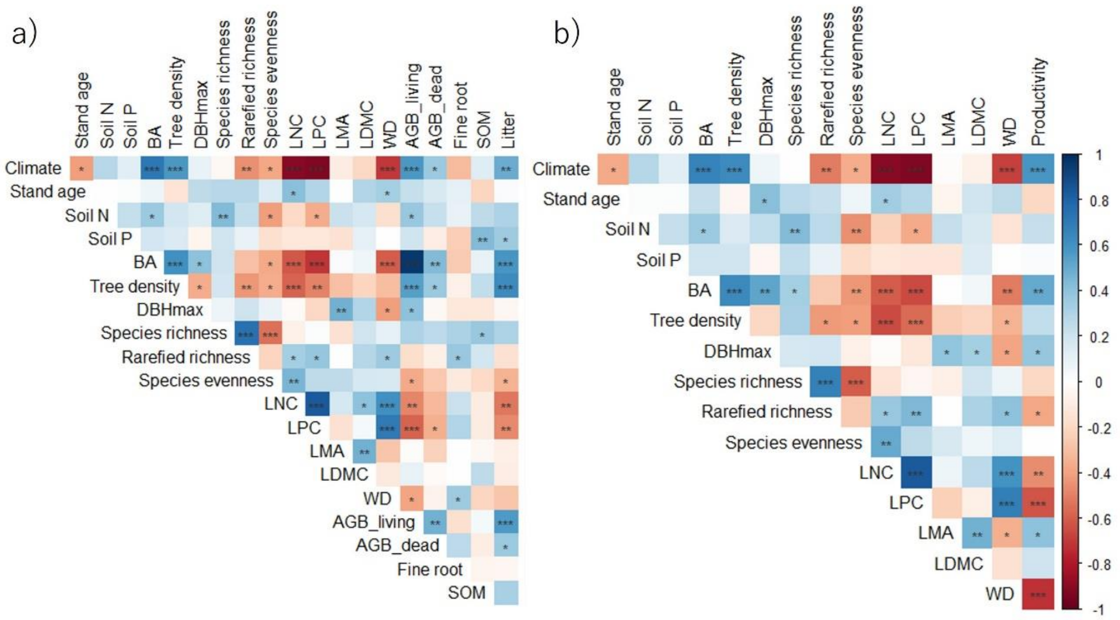

Figure S1: Results of the correlation analyses with the variables that are used in the structural equation models. This is presented for a) total biomass, aboveground living and dead biomass, fine root biomass, soil organic matter, and litter production (the data from 2023), and b) aboveground biomass productivity (the average data between 2022 and 2023). All abbreviations can be found in Appendix S4: Table S2. Pearson pairwise correlation coefficients are given (* $P < 0.05$; ** $P < 0.01$, *** $P < 0.001$).

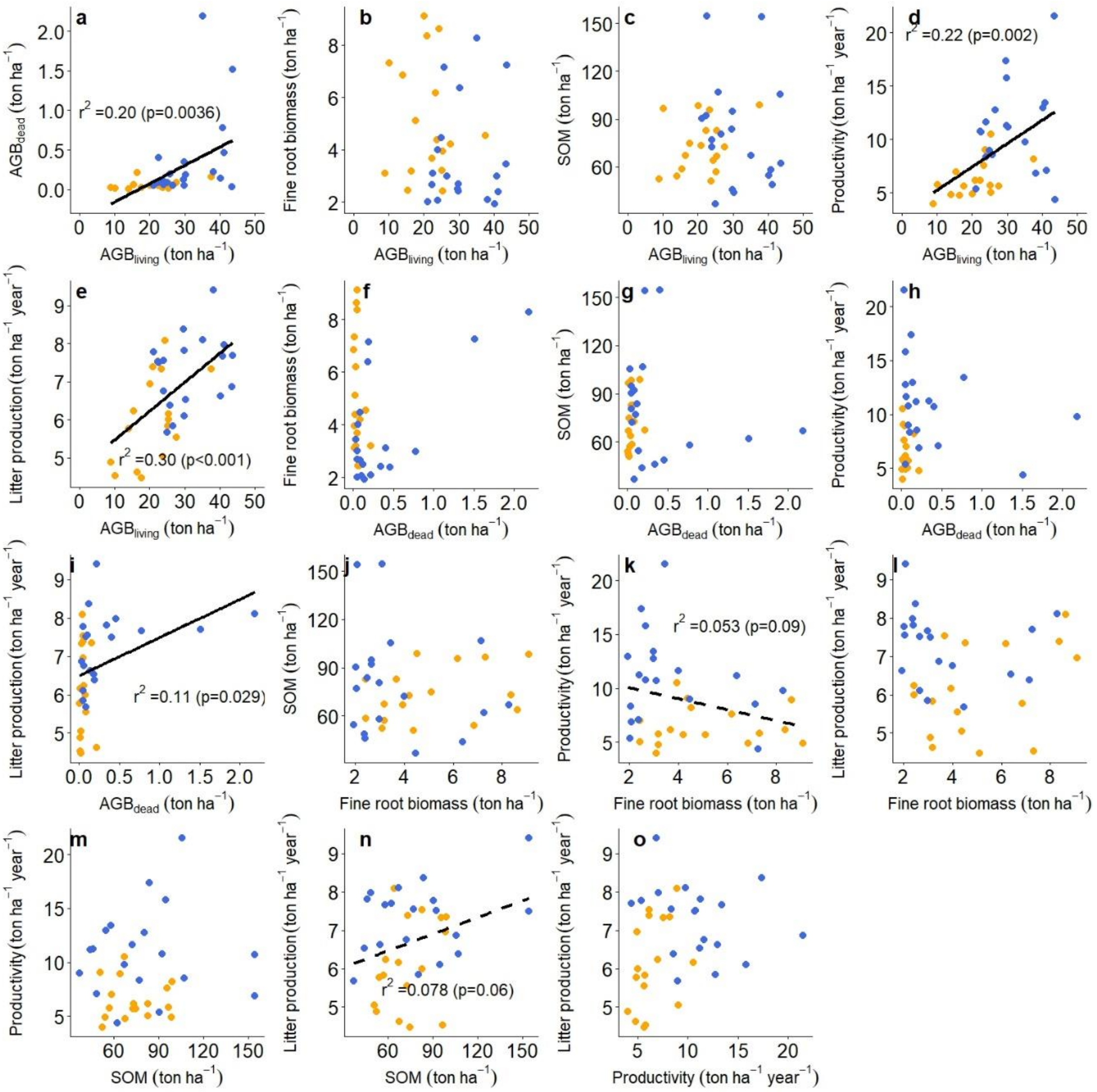

Figure S2: Bivariate relationships of biomass pools and productivity in tropical dry (orange) and wet (blue) forests. The results of a regression line (black line) and a coefficient of determination (r^2: Pearson correlation coefficient) are shown. All abbreviations can be found in Appendix S4: Table S2.
